# Supplementary material for: Targeting NRAS via miR-1304-5p or farnesyltransferase inhibition confers sensitivity to ALK inhibitors in ALK-mutant neuroblastoma
Source: Nat Commun. 2024 Apr 23;15:3422. doi: 10.1038/s41467-024-47771-x (PMC11039739; doi:10.1038/s41467-024-47771-x)
Supplement: Supplementary file 1 — Supplementary Information [file 41467_2024_47771_MOESM1_ESM.pdf]

Supplementary Information

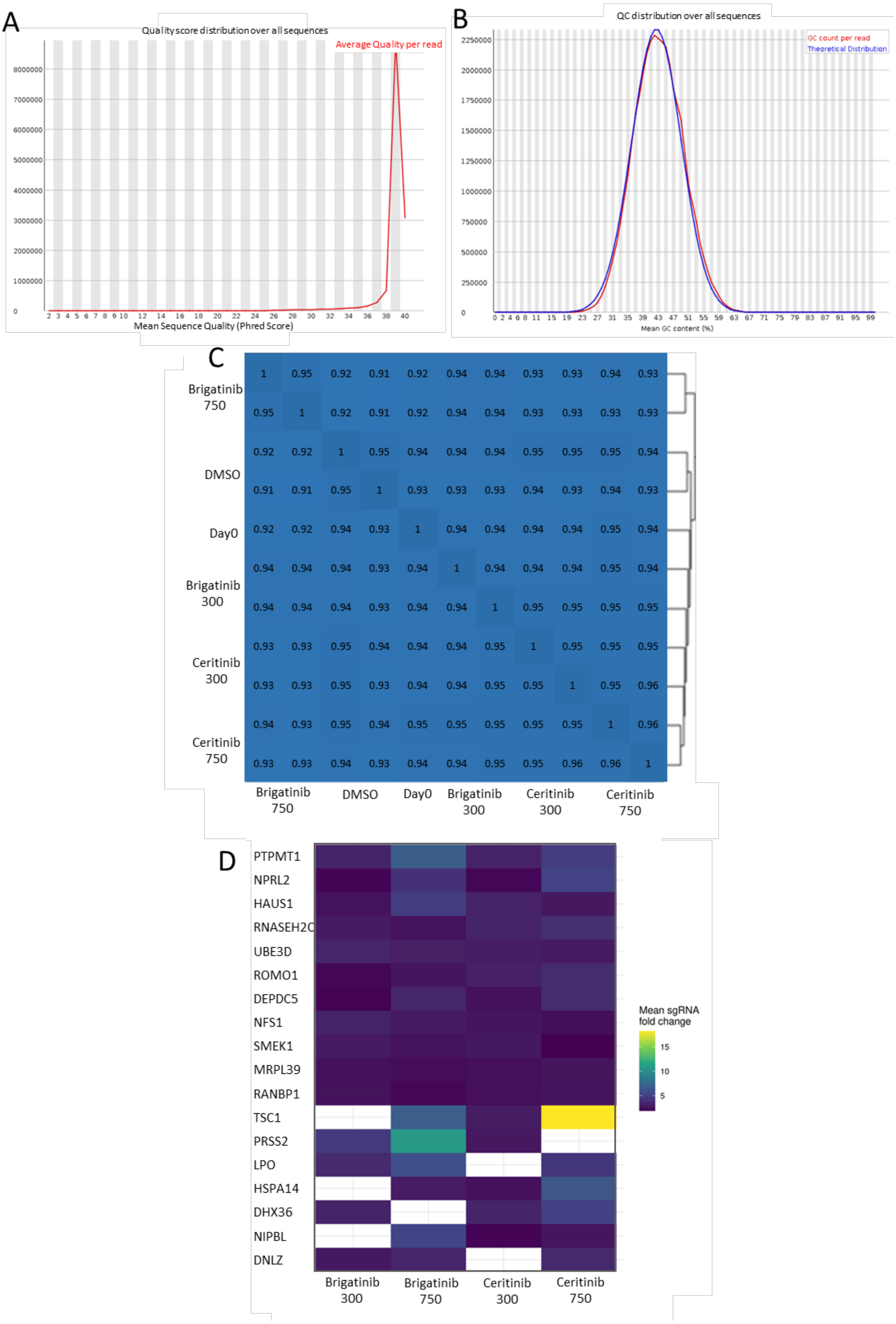
A
Quality score distribution over all sequences
Average Quality per read
Mean Sequence Quality (Phred Score)
B
QC distribution over all sequences
GC count per read
Theoretical Distribution
Mean GC content (%)
C
1 0.95 0.92 0.91 0.92 0.94 0.94 0.93 0.93 0.94 0.93
0.95 1 0.92 0.91 0.92 0.94 0.94 0.93 0.93 0.93 0.93
0.92 0.92 1 0.95 0.94 0.94 0.94 0.95 0.95 0.95 0.94
0.91 0.91 0.95 1 0.93 0.93 0.93 0.94 0.93 0.94 0.93
0.92 0.92 0.94 0.93 1 0.94 0.94 0.94 0.94 0.95 0.94
0.94 0.94 0.94 0.93 0.94 1 0.94 0.94 0.94 0.95 0.94
0.94 0.94 0.94 0.93 0.94 0.94 1 0.95 0.95 0.95 0.95
0.93 0.93 0.95 0.94 0.94 0.94 0.95 1 0.95 0.95 0.95
0.93 0.93 0.95 0.93 0.94 0.94 0.95 0.95 1 0.95 0.96
0.94 0.93 0.95 0.94 0.95 0.95 0.95 0.95 0.95 1 0.96
0.93 0.93 0.94 0.93 0.94 0.94 0.95 0.95 0.96 0.96 1
Brigatinib 750
DMSO
Day0
Brigatinib 300
Ceritinib 300
Ceritinib 750
D
PTPMT1
NPRL2
HAUS1
RNASEH2C
UBE3D
ROMO1
DEPDC5
NFS1
SMEK1
MRPL39
RANBP1
TSC1
PRSS2
LPO
HSPA14
DHX36
NIPBL
DNLZ
Brigatinib 300
Brigatinib 750
Ceritinib 300
Ceritinib 750
Mean sgRNA fold change
15
10
5

Supplementary Figure 1. Quality control and analysis of GECKO screen data

(A) The distribution of per-read 'mean base quality' for each sample (upper plot). All samples from the GeCKO screen overlap with a peak at ~38, which is well beyond the generally acceptable threshold value of 30. (B) The lower plot shows the distribution of the mean GC content of reads per sample. An acceptable mean should be ~40-50% and samples from the same screen should have a similar GC content; the GeCKO samples satisfy those criteria. (C) Pairwise Pearson correlation analysis of all samples from the GeCKO screen. The analysis was performed based on the log-transformed read counts of sgRNAs in each sample. (D) Heatmap of protein-coding gene hits identified in both brigatinib and ceritinib screens (at 300 nM and/or 750 nM of each drug). Coloured squares represent the mean values of fold changes (ALKI TKI versus DMSO treated) of the selected sgRNAs, from lower (blue) to higher (yellow) fold changes.

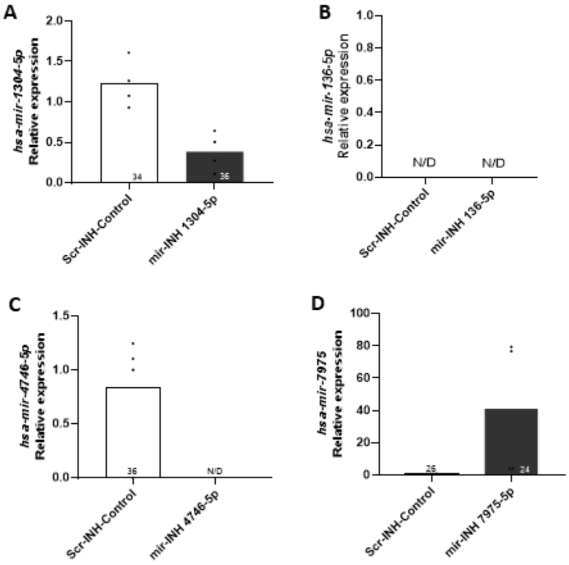

Supplementary Figure 2. ***miR-1304-5p*** **silencing upon transfection with specific miRNA inhibitors**

(A-D) miRNA expression upon transfection with specific miRNA inhibitors assessed by RT-qPCR, relative to the mimic-scrambled control (Scr-INH-Control): *miR-1304-5p* in A, *miR-136-5p* in B, *miR-4746-5p* in C and *miR-7975* in D, the latter using a gene expression assay that cannot discriminate

between -3p and -5p variants. Data points of 2 technical replicates from each of the two biological replicates are shown with columns representing the means. Numbers shown represent the average Ct values. N/D = not detectable. Source data are provided as a Source Data file.

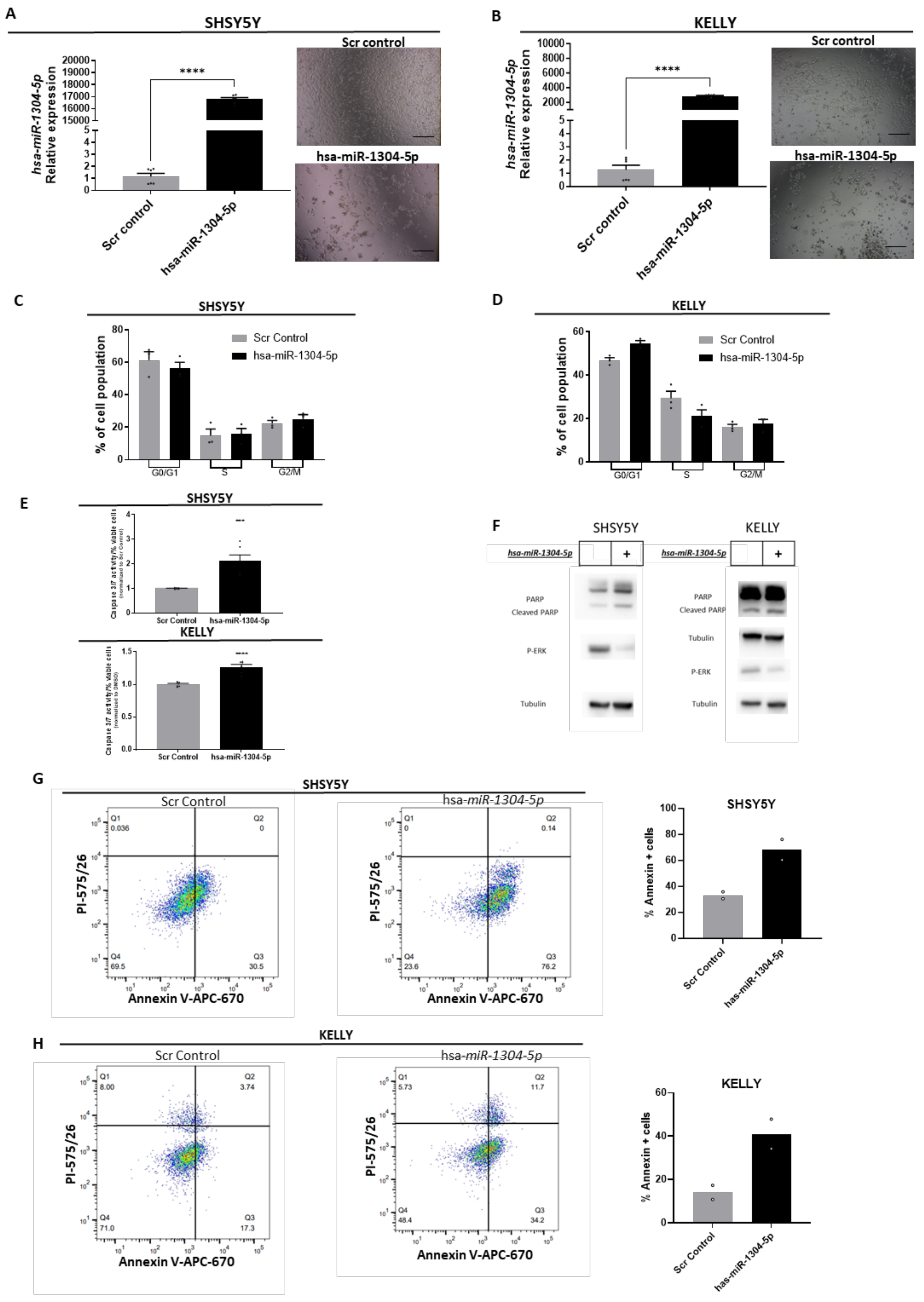
A
SHSY5Y
hsa-miR-1304-5p Relative expression
Scr control
hsa-miR-1304-5p
****
B
KELLY
C
SHSY5Y
% of cell population
Scr Control
hsa-miR-1304-5p
G0/G1
S
G2/M
D
KELLY
E
SHSY5Y
Caspase 3/7 activity/% viable cells
Scr Control
hsa-miR-1304-5p
KELLY
F
SHSY5Y
KELLY
hsa-miR-1304-5p
+
PARP
Cleaved PARP
P-ERK
Tubulin
G
SHSY5Y
Scr Control
hsa-miR-1304-5p
PI-575/26
Annexin V-APC-670
% Annexin + cells
has-miR-1304-5p
H
KELLY

Supplementary Figure 3. miR-1304-5p inhibits NB cell viability inducing apoptosis but not affecting cell cycle progression

(A, B) Confirmation of *miR-1304-5p* upregulation by RT-qPCR upon transfection of the mimic (hsa-miR-1304-5p) relative to the mimic-scrambled control (Scr control) with image inserts showing their effects on cell confluency for the indicated cell lines. Data points of replicates (n=6, 2 technical replicates from three biological replicates) are shown with the columns representing the means ± SEM of the three biological replicates. The black scale bar = 50µm. (C, D) Cell cycle analysis 72 h post-transfection of the indicated cell lines, shown as the percentage of cells in each phase of the cell cycle. Data points from each biological replicate are shown (n=3), and columns and bars represent the means ± SEM of the three biological replicates. (E) Caspase 3/7 activity was measured by luminescence 72 h post-transfection and normalized for the Scr control transfected cells in the indicated cell lines. Data points of replicates (n=6, 2 technical replicates from three biological replicates) are shown with columns representing the means ± SEM of the three biological replicates. (F) Western blot of the indicated proteins 72 h post-transfection of SH-SY5Y or KELLY cells with a *miR-1304-5p* mimic. (G, H) Annexin V (APC) staining shows apoptotic cell fractions (represented in quadrants Q2 + Q3 as Annexin V positive, PI positive or negative) analysed 72 h post-transfection in SHSY5Y and KELLY cells with representative flow cytometry plots shown on the left and quantifications from two biological replicates on the right. Data points from each biological replicate are shown (n=2). Statistical comparison was conducted using a one-way Anova with Tukey`s post-test in C and D and a Two-tailed Student`s t-test in A, B, E (A)****$p<10^{-15}$. (B)****$p=3.8x10^{-11}$. (E) ***p= 0.00058, ****p=0.000089. Source data are provided as a Source Data file.

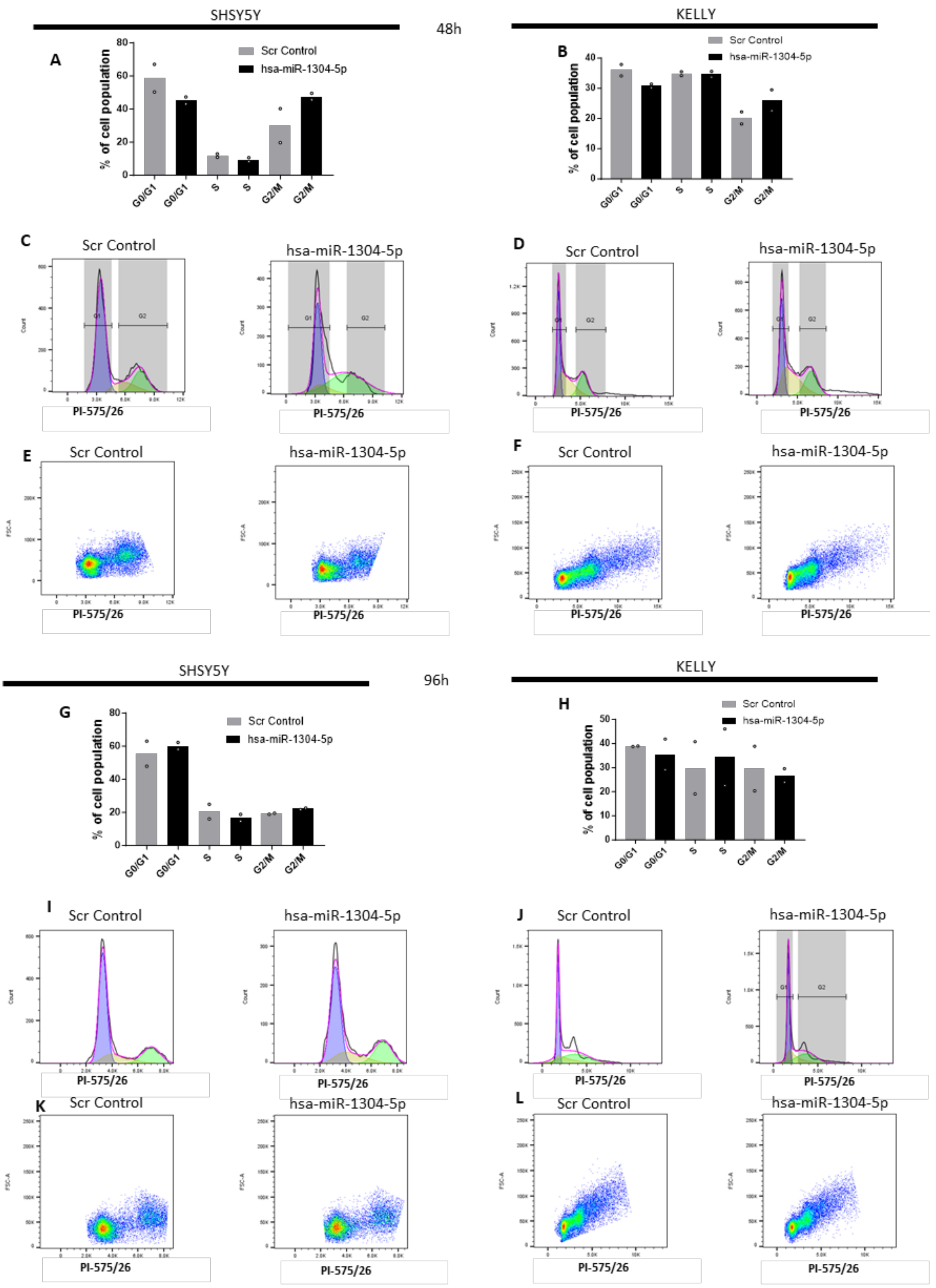

Supplementary Figure 4. Ectopic overexpression of miR-1304-5p does not affect the cell cycle 48h and 96h post-transfection

Cell cycle profiles (indicated by PI incorporation into the DNA of permeabilised cells) were determined 48 hours (A-F) or 96 hours (G-L) post-transfection with a *miR-1304-5p* mimic. (A-F) Data points from

two biological replicates are shown (n=2) for SHSY5Y (A) and KELLY (B) cells with representative cell cycle flow cytometry profiles (SHSY5Y in C and KELLY in D) and cell populations (SHSY5Y in E and KELLY in F) shown 48h post-transfection. (G-L) Percentage means of two biological replicates are shown for SHSY5Y (G) and KELLY (H) cells with representative cell cycle flow cytometry profiles (SHSY5Y in I and KELLY in J) and cell populations (SHSY5Y in K and KELLY in L), shown 96h post-transfection. Source data are provided as a Source Data file.

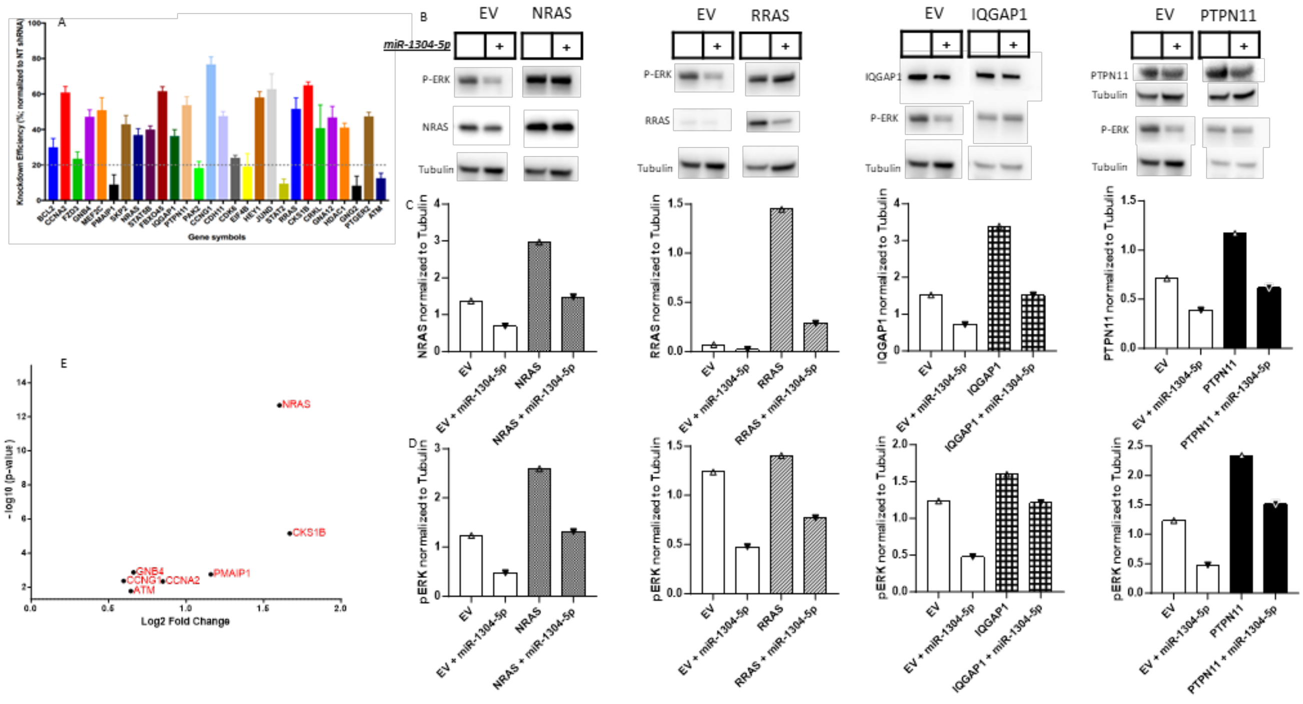

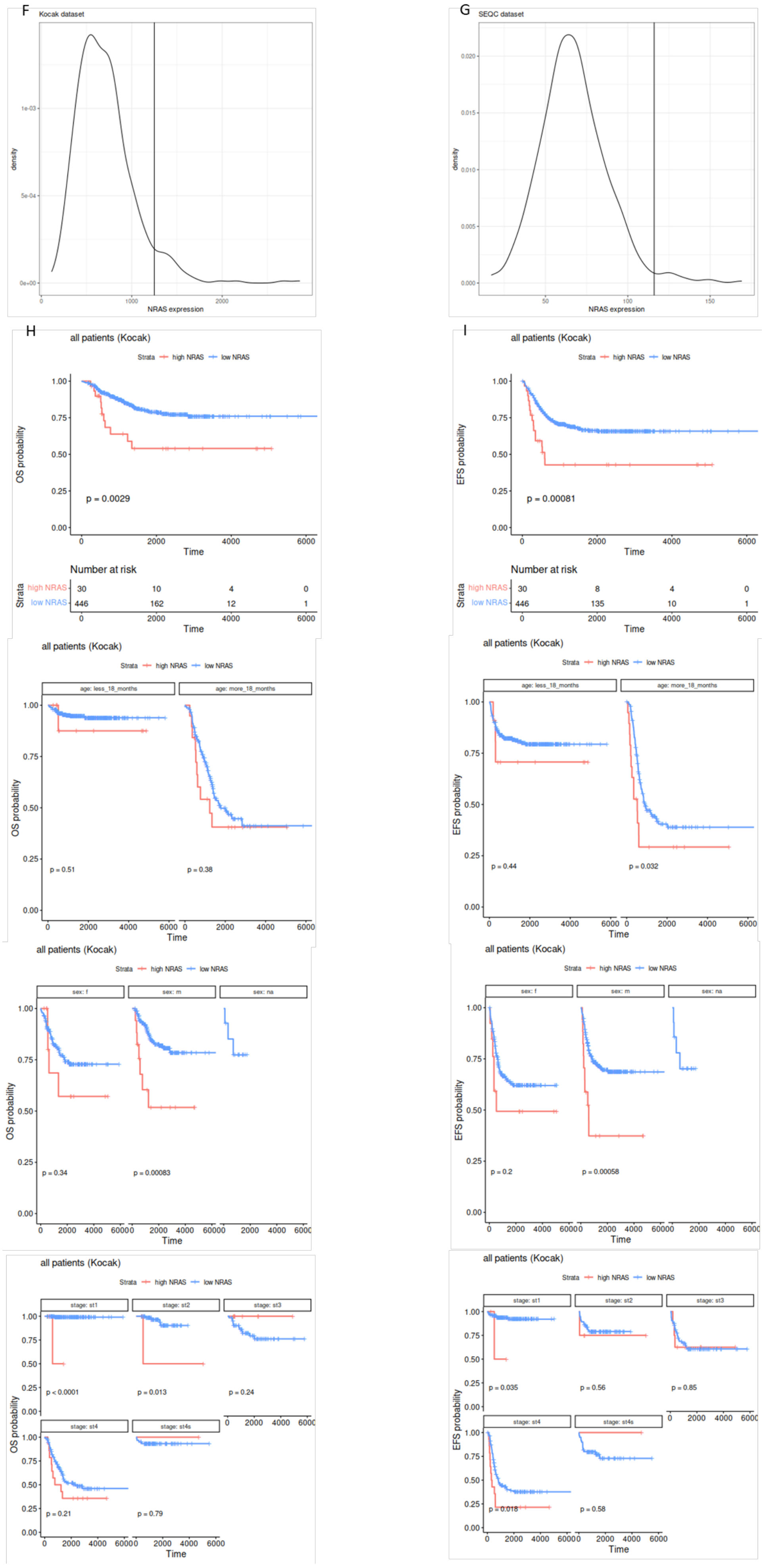
F
Kocak dataset
density
NRAS expression
G
SEQC dataset
density
NRAS expression
H
all patients (Kocak)
Strata high NRAS low NRAS
OS probability
p = 0.0029
Time
Number at risk
Strata
high NRAS 30 10 4 0
low NRAS 446 162 12 1
I
all patients (Kocak)
Strata high NRAS low NRAS
EFS probability
p = 0.00081
Time
Number at risk
Strata
high NRAS 30 8 4 0
low NRAS 446 135 10 1
all patients (Kocak)
age: less_18_months
age: more_18_months
OS probability
p = 0.51
p = 0.38
Time
all patients (Kocak)
age: less_18_months
age: more_18_months
EFS probability
p = 0.44
p = 0.032
Time
all patients (Kocak)
sex: f
sex: m
sex: na
OS probability
p = 0.34
p = 0.00083
Time
all patients (Kocak)
sex: f
sex: m
sex: na
EFS probability
p = 0.2
p = 0.00058
Time
all patients (Kocak)
stage: st1
stage: st2
stage: st3
stage: st4
stage: st4s
OS probability
p < 0.0001
p = 0.013
p = 0.24
p = 0.21
p = 0.79
Time
all patients (Kocak)
stage: st1
stage: st2
stage: st3
stage: st4
stage: st4s
EFS probability
p = 0.035
p = 0.56
p = 0.85
p = 0.018
p = 0.58
Time

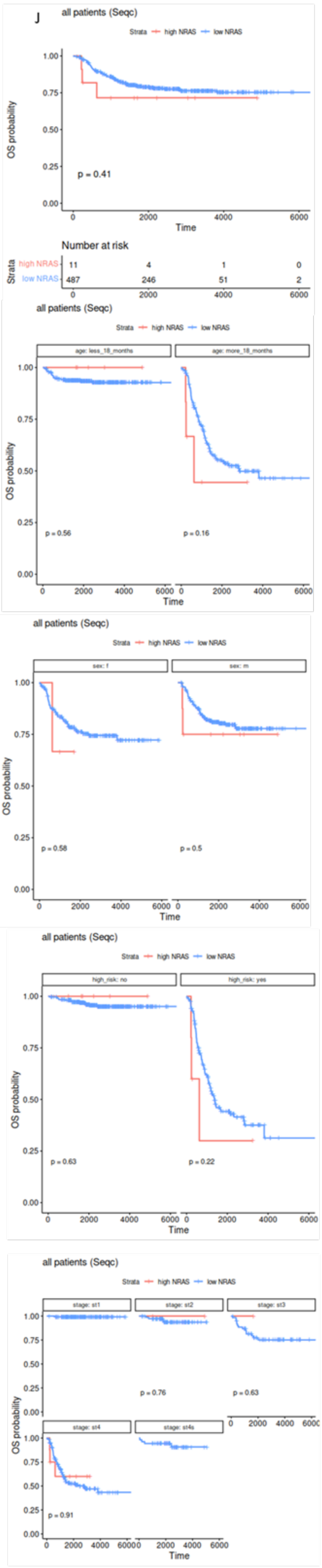

J
all patients (Seqc)
Strata high NRAS low NRAS
OS probability
p = 0.41
Time
Number at risk
high NRAS 11 4 1 0
low NRAS 487 246 51 2
age: less_18_months
age: more_18_months
p = 0.56
p = 0.16
sex: f
sex: m
p = 0.58
p = 0.5
high_risk: no
high_risk: yes
p = 0.63
p = 0.22
stage: st1
stage: st2
stage: st3
stage: st4
stage: st4s
p = 0.76
p = 0.63
p = 0.91

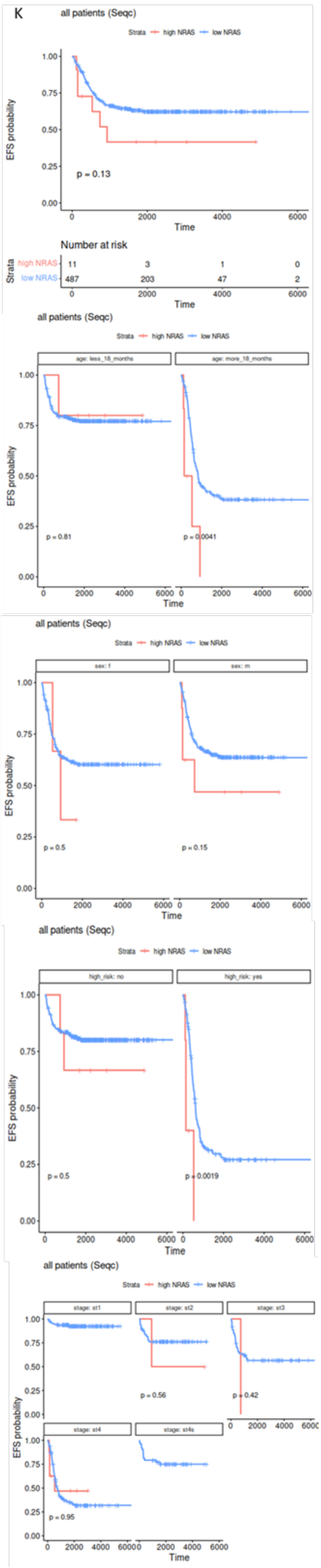

K
all patients (Seqc)
Strata high NRAS low NRAS
EFS probability
p = 0.13
Time
Number at risk
high NRAS 11 3 1 0
low NRAS 487 203 47 2
age: less_18_months
age: more_18_months
p = 0.81
p = 0.0041
sex: f
sex: m
p = 0.5
p = 0.15
high_risk: no
high_risk: yes
p = 0.5
p = 0.0019
stage: st1
stage: st2
stage: st3
stage: st4
stage: st4s
p = 0.56
p = 0.42
p = 0.95

Supplementary Figure 5. Identification and validation of *miR-1304-5p* target genes

(A) RT-qPCR validation of the 28 target genes associated with cancer pathways predicted to be dysregulated by *miR-1304-5p* from expression microarray results, and predicted to be direct targets of *miR-1304-5p* by TargetScan 7.1. Gene expression is represented as the knockdown efficiency compared to a scrambled negative control (Scr control). The dotted line reflects the 20% knockdown level threshold applied. (B-D) Western blot (B) and quantification (with ImageJ, C-D) of the indicated proteins at day 6 post-transduction with the cDNA of the 4 target genes (also containing the 3'UTR regions of each gene) +/- co-transfection of a *miR-1304-5p* mimic into SHSY5Y cells. Beta-Tubulin and pERK blots for the EV control are replicated in each of the panels for easier visualisation against the corresponding overexpression sample (NRAS, RRAS, IQGAP1 and PTPN11). Data are representative of n=2. (E) Microarray detected expression of the 28 genes identified as potential targets of *miR-1304-5p* in ceritinib resistant (n=2) versus sensitive (n=2) orthotopic NB xenograft models [34]. Hits shown have a fold change > 1 ($\log_2$=0) and p<0.05 (-$\log_{10}$=1.3). (F-G) Density plots of the patient populations described in the Kocak (F) and SEQC (G) datasets, based on NRAS expression. (H-K) Expression of NRAS mRNA measured in 470 (Kocak, H-I) and 498 (SEQC, J-K) NB patients. From left to right the panels show OS (H) or EFS (I) in all patients, stratified by age (>18 months or <18 months), sex or stage in the Kocak dataset. From left to right, the panels show OS (H) or EFS (I) in all patients, stratified by age, sex, risk-group or stage in the SEQC dataset. To note, there were no patients of exactly 18 months of age. Log-rank test, with p values specified in the figure panels above (significance considered when p<0.05). Source data are provided as a Source Data file.

A

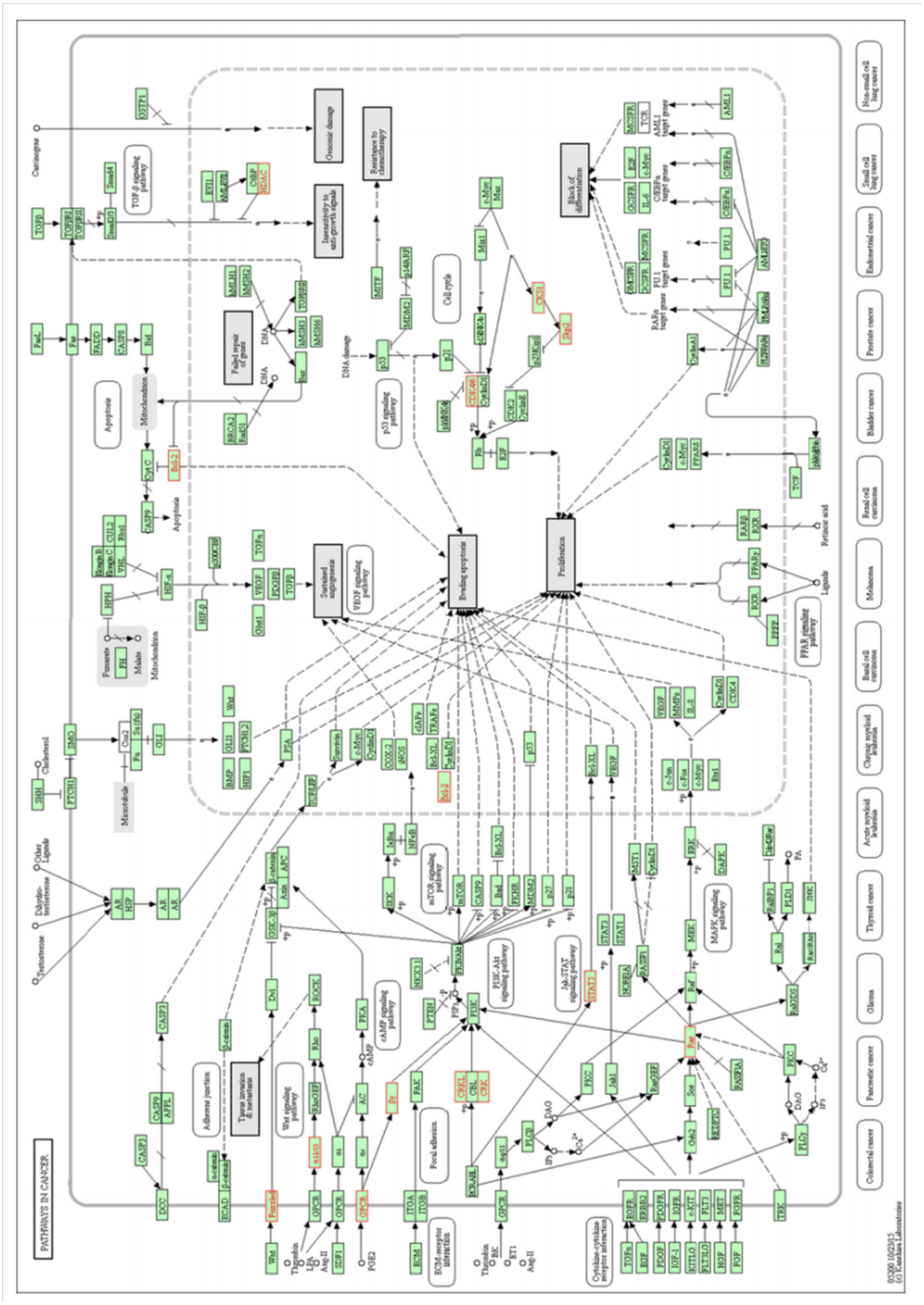

B

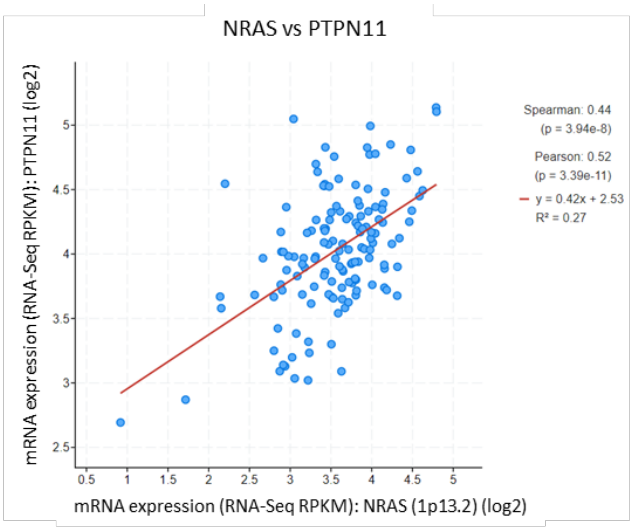

NRAS vs PTPN11
Spearman: 0.44
(p = 3.94e-8)
Pearson: 0.52
(p = 3.39e-11)
y = 0.42x + 2.53
R² = 0.27
mRNA expression (RNA-Seq RPKM): PTPN11 (log2)
mRNA expression (RNA-Seq RPKM): NRAS (1p13.2) (log2)

C

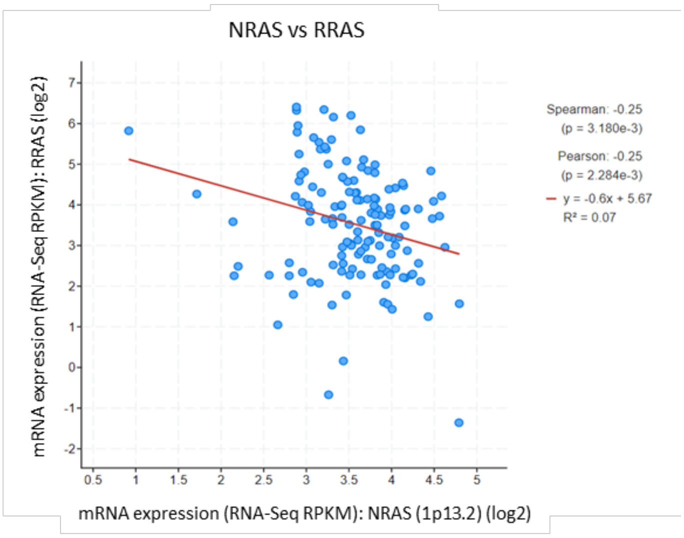

NRAS vs RRAS
Spearman: -0.25
(p = 3.180e-3)
Pearson: -0.25
(p = 2.284e-3)
y = -0.6x + 5.67
R² = 0.07
mRNA expression (RNA-Seq RPKM): RRAS (log2)
mRNA expression (RNA-Seq RPKM): NRAS (1p13.2) (log2)

D

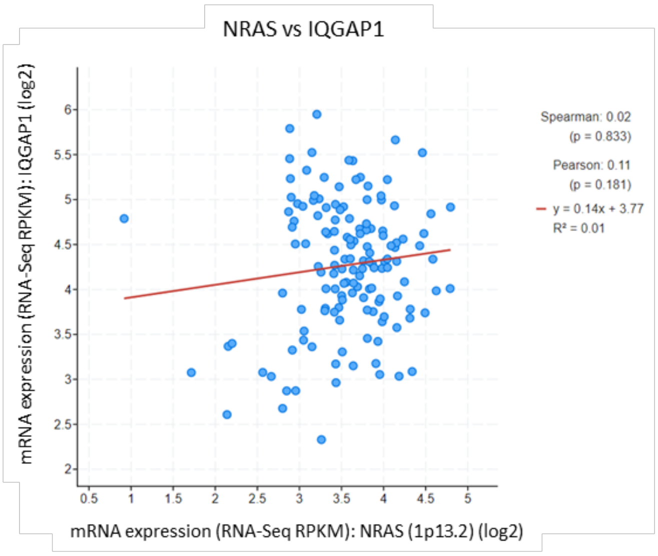

NRAS vs IQGAP1
Spearman: 0.02
(p = 0.833)
Pearson: 0.11
(p = 0.181)
y = 0.14x + 3.77
R² = 0.01
mRNA expression (RNA-Seq RPKM): IQGAP1 (log2)
mRNA expression (RNA-Seq RPKM): NRAS (1p13.2) (log2)

Supplementary Figure 6. KEGG pathway analysis and correlation of expression of the top *miR-1304-5p* target genes in clinical samples

(A) KEGG pathways associated with cancer as determined by the 276 genes selected from microarray and binding prediction analyses. (B-D) Correlation of bulk RNA sequencing expression levels of *NRAS* with *PTPN11*, *RRAS* or *IQGAP1* in 143 NB patient tumours [33] shown as a $\log_2$ scale.

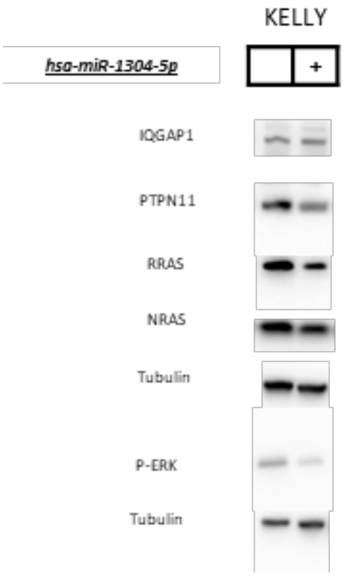

Supplementary Figure 7. Western blot of the indicated proteins 72 h post-transfection of KELLY cells with a *miR-1304-5p* mimic.

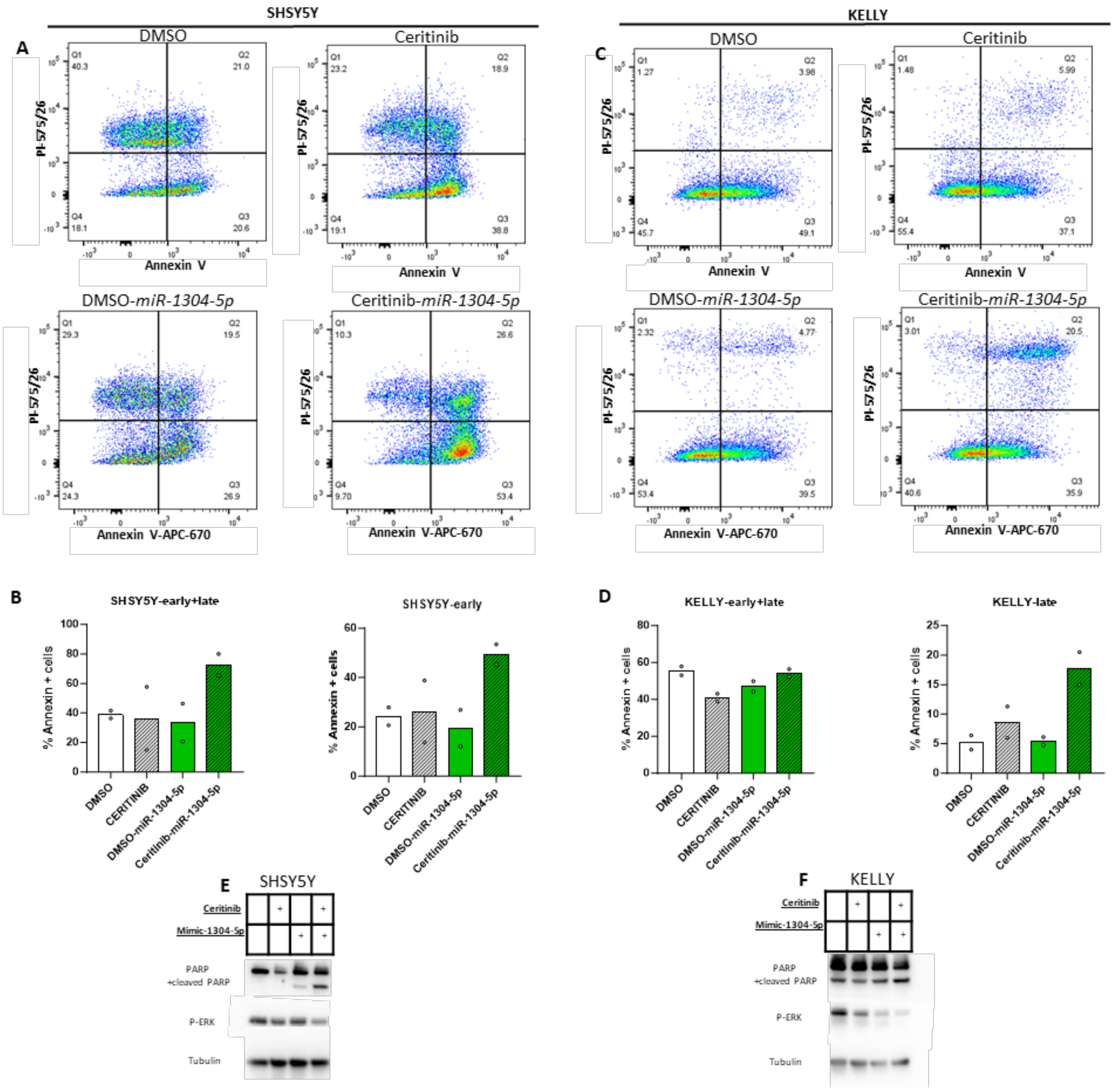

Supplementary Figure 8. *The miR-1304-5p* mimic stimulates and enhances apoptosis when used in combination with an ALK TKI.

(**A-D**) Annexin V (APC) staining shows apoptotic cell fractions analysed 48h after transfection with a *miR-1304-5p* mimic with an additional 72 hours of treatment with an ALK TKI (ceritinib, 100nM concentration) in SHSY5Y (A-B) and KELLY cells (C-D) with cell populations shown in A and C and quantifications in B and D, with data points from each replicate shown from two biological replicates. (**E-F**) Western blot of the indicated proteins analysed 48h following transfection with a *miR-1304-5p* mimic with an additional 72 hours of treatment with an ALK TKI (ceritinib, 100nM concentration) in

SHSY5Y (E) and KELLY cells (F). Early apoptosis = Annexin V positive, PI negative (quadrant Q3); late apoptosis = Annexin V and PI positive (quadrant Q2). Source data are provided as a Source Data file.

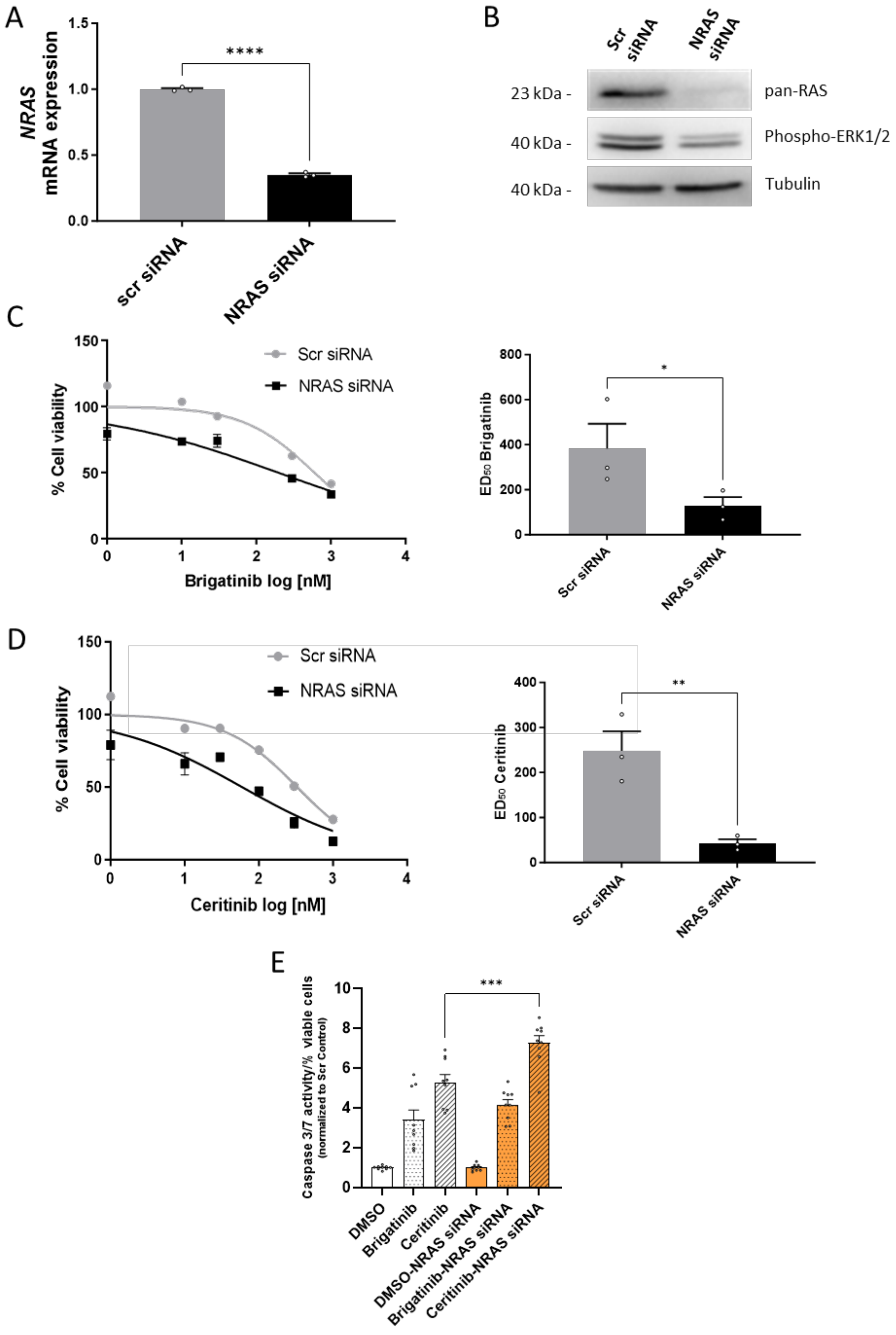

Supplementary Figure 9. *NRAS* knockdown increases NB sensitivity to ALK TKIs.

(A) Transcript levels of *NRAS* following siRNA-mediated knockdown in SHSY-5Y cells detected by RT-qPCR relative to the siRNA-scrambled control (Scr siRNA). (B) Western blot of the indicated proteins

in SHSY5Y cells upon NRAS knockdown (shown in A). (C-D) SH-SY5Y cell viability (measured via CTG) and ED_{50} following a combination of NRAS siRNA transfection and brigatinib (C) or ceritinib (D) treatment for 72 h at the indicated doses. ED_{50} values shown in the graphs on the right were calculated from the non-linear fit curves shown on the left. Data points shown are representative of three biological replicates. Statistical comparisons were conducted with a two-way ANOVA with Sidak`s post-test and Student`s t-test of means ± SEM. *p<0.05, **p<0.01. (E) Apoptosis determined by caspase 3/7 activity in SH-SY5Y cells treated with a combination of a NRAS siRNA and brigatinib (1000nM) or ceritinib (1000nM). Data points of technical replicates (n=9 representing three technical replicates from the three biological replicates) are shown with the means and SEMs of the biological replicates represented by the columns and error bars. Significance was determined using a one-way ANOVA with Tukey`s post-test of the means of the biological replicates. ***p=0.00096. Source data are provided as a Source Data file.

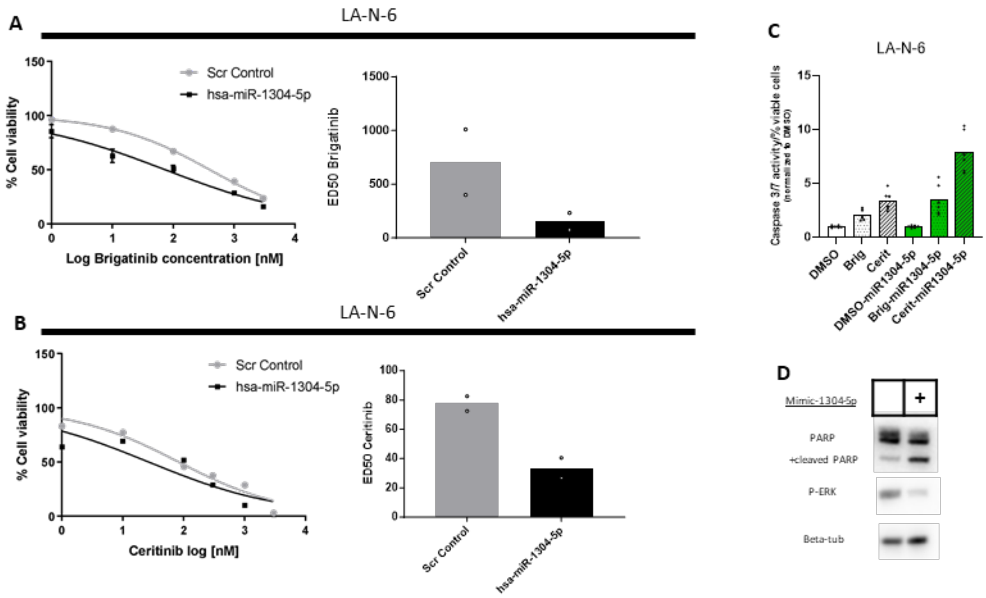

Supplementary Figure 10. The *miR-1304-5p* mimic is a therapeutic target when used in combination with ALK TKIs in the LA-N-6 (ALK kinase domain WT) neuroblastoma cell line

(A-B) LA-N-6 cell viability (measured via CTG assay) and ED_{50} upon transfection of a *miR-1304-5p* mimic and treatment with brigatinib (A) or ceritinib (B) for 72 h at the indicated doses. ED50 values shown in the graphs on the right were calculated from the non-linear fit curves on the left. Data points shown

(n= 2) are representative of two independent biological replicates. (C) Apoptosis determined by caspase 3/7 activity in LA-N-6 cells treated with a combination of a *miR-1304-5p* mimic and brigatinib (1000nM) or ceritinib (1000nM). Data points shown (n=6 technical replicates, 3 from each of 2 biological replicates) with columns showing the means of the biological replicates. (D) Western blot of the indicated proteins 72 h post-transfection of LA-N-6 with a *miR-1304-5p* mimic. Source data are provided as a Source Data file.

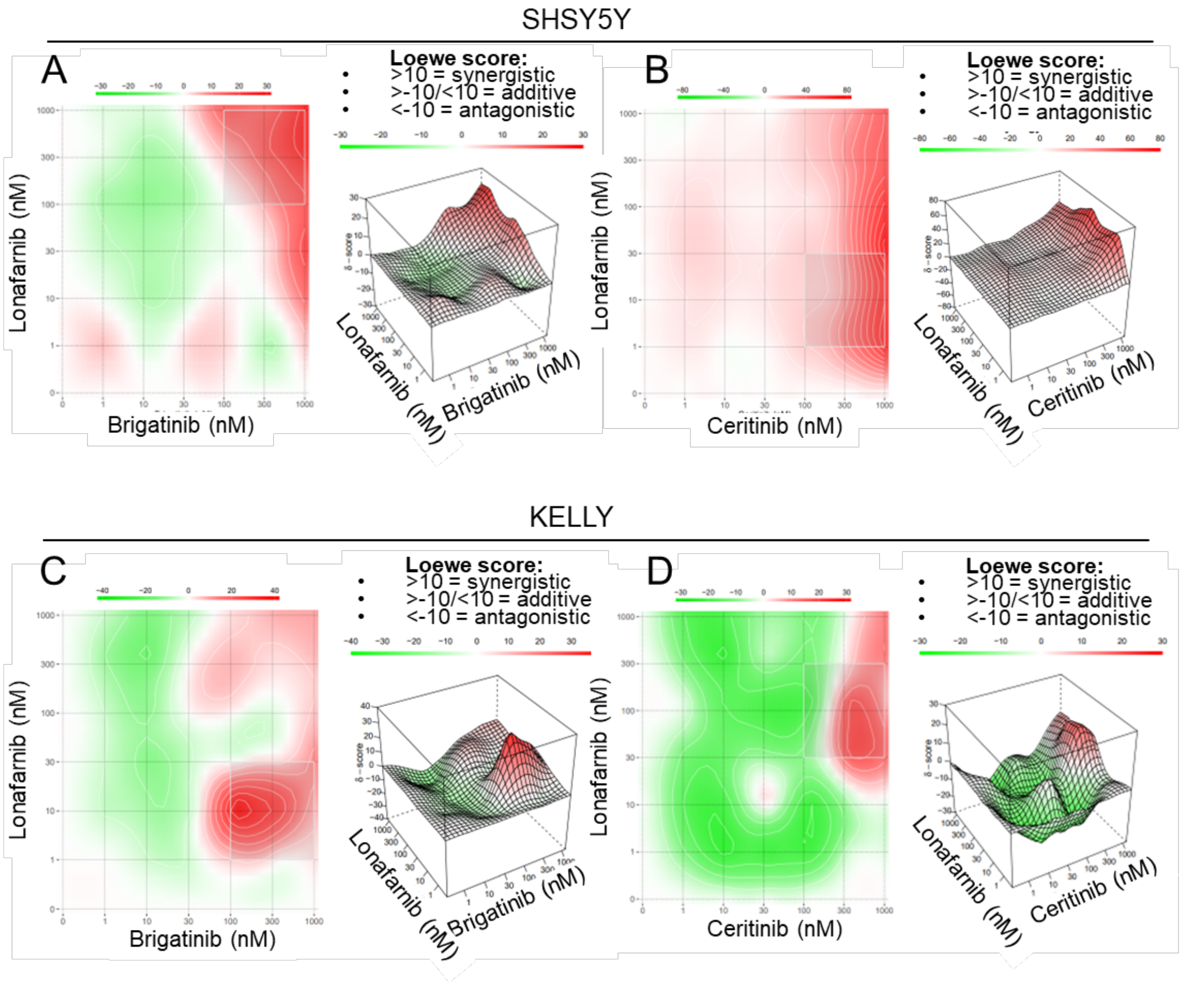

Supplementary Figure 11. Two-dimensional and three-dimensional representation of ALK TKI and lonafarnib synergism in NB cells

Loewe synergy scores calculated with the Synergy Finder tool[85] for SH-SY5Y cells treated with a combination of lonafarnib and brigatinib (A) or lonafarnib and ceritinib (B) and for KELLY cells treated with lonafarnib and brigatinib (C) or lonafarnib and ceritinib (D). Data are shown as both 2D and 3D

models and are represented as color gradients from antagonistic (darker green, loewe score <-10) to synergistic (darker red, loewe score >10). Synergy scores >- 10 and <10 are considered additive.

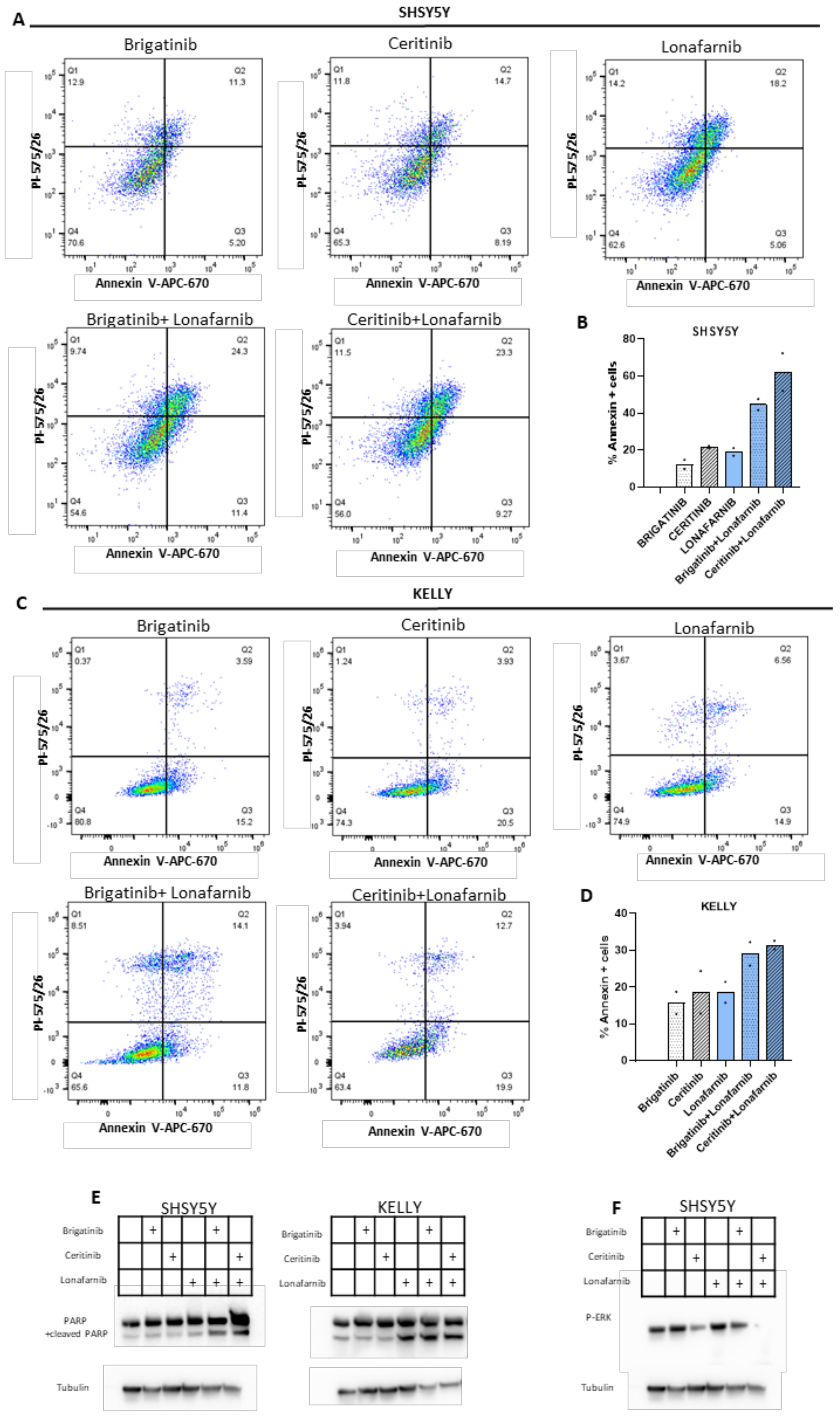

Supplementary Figure 12. A combination of ALK TKIs and the FTI lonafarnib increases apoptosis measured via Annexin V positivity and PARP cleavage in NB cells.

(**A-D**) Annexin V (APC) staining shows apoptotic cell fractions analysed after 72h of co-treatment with an ALK TKI (brigatinib or ceritinib, both at 100uM) with an FTI (lonafarnib, 1000uM) in SHSY5Y (A-B) and KELLY cells (C-D) with cell populations shown in A and C and quantifications (on the Annexin V positive population, either PI positive or negative, in quadrants Q2 + Q3) in B and D from two biological replicates. Data points in B and D represent two independent biological replicates. (**E-F**) Western blots of the indicated proteins analysed after 72h of co-treatment with an ALK TKI (brigatinib or ceritinib, both at 100uM) and an FTI (lonafarnib, 1000uM) in SHSY5Y and KELLY cells. Source data are provided as a Source Data file.

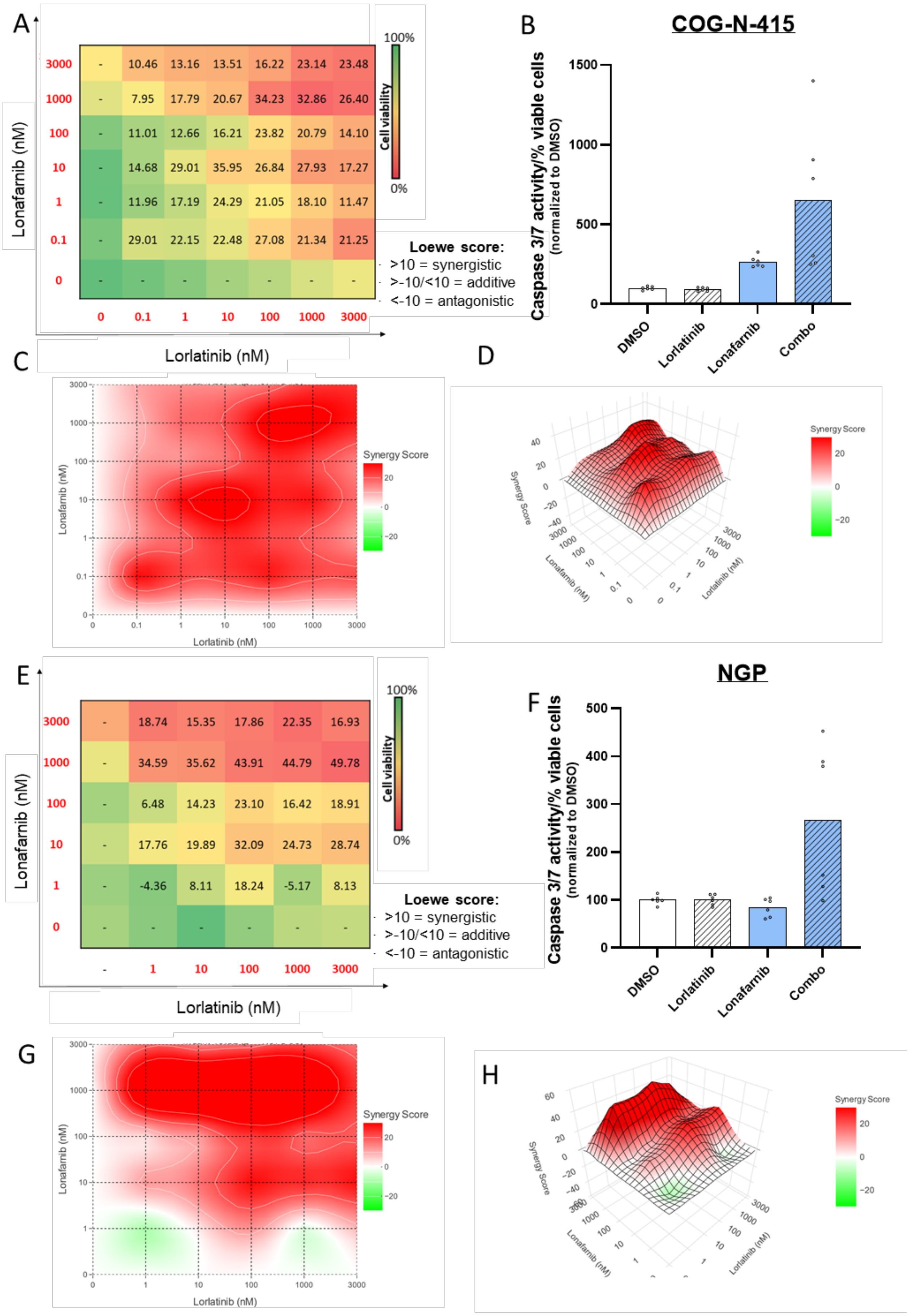
A
Lonafarnib (nM)
3000 - 10.46 13.16 13.51 16.22 23.14 23.48
1000 - 7.95 17.79 20.67 34.23 32.86 26.40
100 - 11.01 12.66 16.21 23.82 20.79 14.10
10 - 14.68 29.01 35.95 26.84 27.93 17.27
1 - 11.96 17.19 24.29 21.05 18.10 11.47
0.1 - 29.01 22.15 22.48 27.08 21.34 21.25
0 - - - - - - -
0 0.1 1 10 100 1000 3000
Lorlatinib (nM)
100%
Cell viability
0%
Loewe score:
>10 = synergistic
>-10/<10 = additive
<-10 = antagonistic
B
COG-N-415
Caspase 3/7 activity/% viable cells
(normalized to DMSO)
1500
1000
500
0
DMSO
Lorlatinib
Lonafarnib
Combo
C
Lonafarnib (nM)
Lorlatinib (nM)
Synergy Score
D
Synergy Score
Lonafarnib (nM)
Lorlatinib (nM)
E
Lonafarnib (nM)
3000 - 18.74 15.35 17.86 22.35 16.93
1000 - 34.59 35.62 43.91 44.79 49.78
100 - 6.48 14.23 23.10 16.42 18.91
10 - 17.76 19.89 32.09 24.73 28.74
1 - -4.36 8.11 18.24 -5.17 8.13
0 - - - - - -
- 1 10 100 1000 3000
Lorlatinib (nM)
100%
Cell viability
0%
Loewe score:
>10 = synergistic
>-10/<10 = additive
<-10 = antagonistic
F
NGP
Caspase 3/7 activity/% viable cells
(normalized to DMSO)
500
400
300
200
100
0
DMSO
Lorlatinib
Lonafarnib
Combo
G
Lonafarnib (nM)
Lorlatinib (nM)
Synergy Score
H
Synergy Score
Lonafarnib (nM)
Lorlatinib (nM)

Supplementary Figure 13. A combination of the ALK inhibitor lorlatinib and the FTI lonafarnib act synergistically in ALK mutant PDX and an ALK WT cell line via induction of apoptosis.

(**A**) Dose-response matrix of lonafarnib (0.1-3000nM) and lorlatinib (0.1-3000nM) alone or in combination, following 72 h incubation with COG-N-415 (ALK mutant, MYCN amplified) PDX cells. Loewe synergy scores (Synergy Finder) and cell viability (CTG) results from two biological replicates are shown. Colour gradients: % cell viability normalised to DMSO (from green: 100%, to red: 0%). Scores >10 represent synergism. (**B**) Apoptosis (caspase 3/7 activity per cell population normalized to DMSO treated cells) of COG-N-415 cells treated with a combination of lonafarnib (1µM) and lorlatinib (1µM), or single agents (same doses), for 72 h. Data points shown (n= 6) are from two biological replicates (each with three technical replicates). (**C-D**) Loewe synergy scores calculated with the Synergy Finder tool [85] for COG-N-415 PDX cells treated with a combination of lonafarnib and lorlatinib shown as both 2D (C) and 3D (D) models and are represented as colour gradients from antagonistic (darker green, loewe score <-10) to synergistic (darker red, loewe score >10). Synergy scores >- 10 and <10 are considered additive. (**E**) Dose-response matrix of lonafarnib (1-3000nM) and lorlatinib (1-3000nM) alone or in combination, following 72 h incubation with NGP (ALK WT, MYCN amplified) cells. Loewe synergy scores (Synergy Finder) and cell viability (CTG) results from two biological replicates are shown. Colour gradients: % cell viability normalised to DMSO (from green: 100%, to red: 0%). Scores >10 represent synergism. (**F**) Apoptosis (caspase 3/7 activity per cell population normalized to DMSO treated cells) of NGP cells treated with a combination of lonafarnib (1µM) and lorlatinib (1µM), or single agents (same doses), for 72 h. Data points shown (n= 6) are from two biological replicates (each with three technical replicates). (**G-H**) Loewe synergy scores calculated with the Synergy Finder tool [85] for NGP cells treated with a combination of lonafarnib and lorlatinib shown as both 2D (G) and 3D (H) models and are represented as color gradients from antagonistic (darker green, loewe score <-10) to synergistic (darker red, loewe score >10). Synergy scores >- 10 and <10 are considered additive. Source data are provided as a Source Data file.

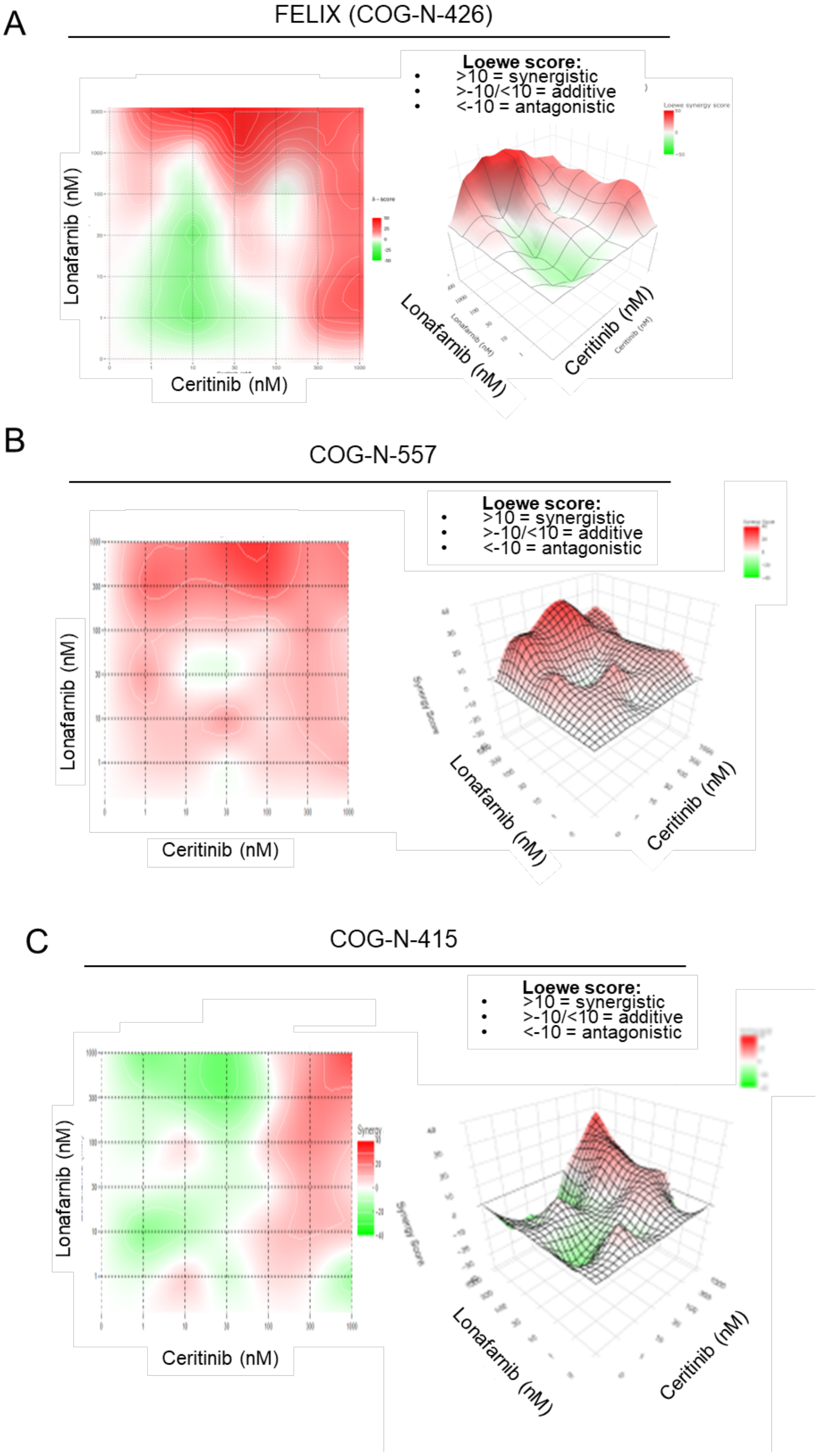
A
FELIX (COG-N-426)
Loewe score:
>10 = synergistic
>-10/<10 = additive
<-10 = antagonistic
Lonafarnib (nM)
Ceritinib (nM)
B
COG-N-557
Loewe score:
>10 = synergistic
>-10/<10 = additive
<-10 = antagonistic
Lonafarnib (nM)
Ceritinib (nM)
C
COG-N-415
Loewe score:
>10 = synergistic
>-10/<10 = additive
<-10 = antagonistic
Lonafarnib (nM)
Ceritinib (nM)

Supplementary Figure 14. Two-dimensional and three-dimensional representation of ALK TKI and lonafarnib synergism in NB PDX cells

Loewe synergy scores calculated with the Synergy Finder tool [85] for FELIX (COG-N-426) (A)**,** COG-N-557 (B) and for COG-N-415 (C) cells treated with lonafarnib and ceritinib for 72 h. Data are shown as both 2D and 3D models and are represented as color gradients from antagonistic (darker green, loewe score <-10) to synergistic (darker red, loewe score >10). Synergy scores >- 10 and <10 are considered additive.

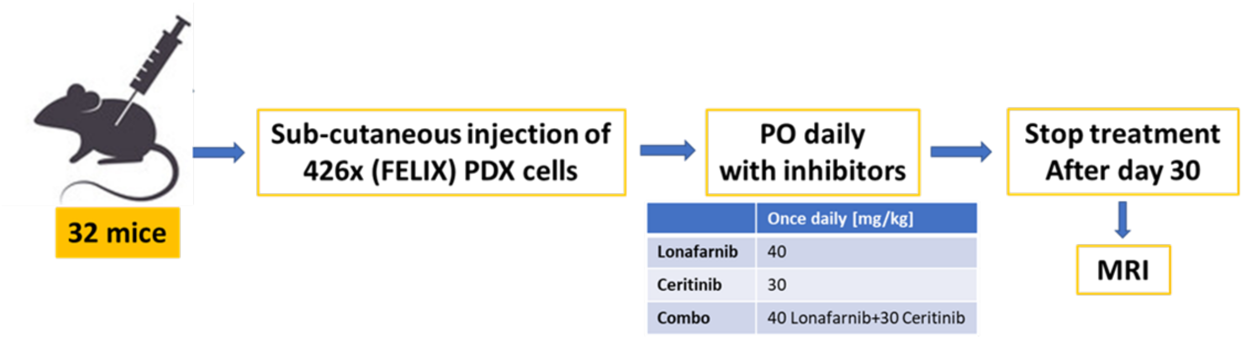

Supplementary Figure 15. Flow chart of the *in vivo* study

Flow chart of the *in vivo* study of ceritinib and lonafarnib treatment of FELIX (COG-N-426x) PDX. PO = oral gavage; MRI = magnetic resonance imaging. The same procedure was followed for the *in vivo* study using COG-N-415x PDX.

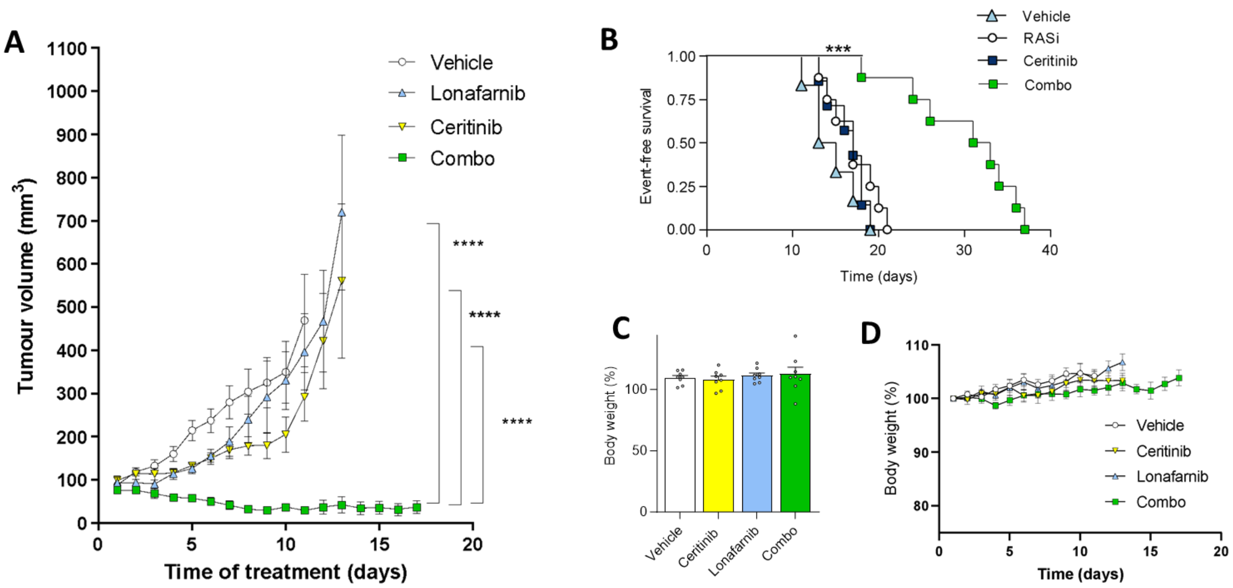

Supplementary Figure 16. A combination of an ALK inhibitor (ceritinib) with an FTI (lonafarnib) significantly reduces MYCN amplified PDX tumour growth *in vivo*

(A) Tumour volume over time of NSG mice injected sub-cutaneously with COG-N-415x primary NB cells which reached 75mm^3 before daily administration of either vehicle (20% hydroxypropyl beta cyclodextrin), ceritinib (30 mg/kg), lonafarnib (40 mg/kg), or ceritinib and lonafarnib (combo, same concentrations as used for the single agent arms). The study endpoint was reached when tumours became 15 mm in diameter or following 30 days of treatment, whichever came first. Data points (n=8 individual animals) represent means ± SEM, shown until the experimental endpoint (as defined above) of the first animal within each treatment group. (B) Kaplan–Meier EFS analysis. ***p<0.001 (Log-rank test). (C) Mouse body weight at the experimental endpoint relative to baseline weights for each treatment group. Data points (n=8) represent each animal`s weight with means ± SEM. One-way ANOVA with Tukey`s post-test determined significance at each experimental endpoint in A and C. ****p<0.0001. (D) Mouse body weight recorded every day of treatment relative to baseline weights for each treatment group. Data points (n=8) represent means ± SEM. Source data are provided as a Source Data file.

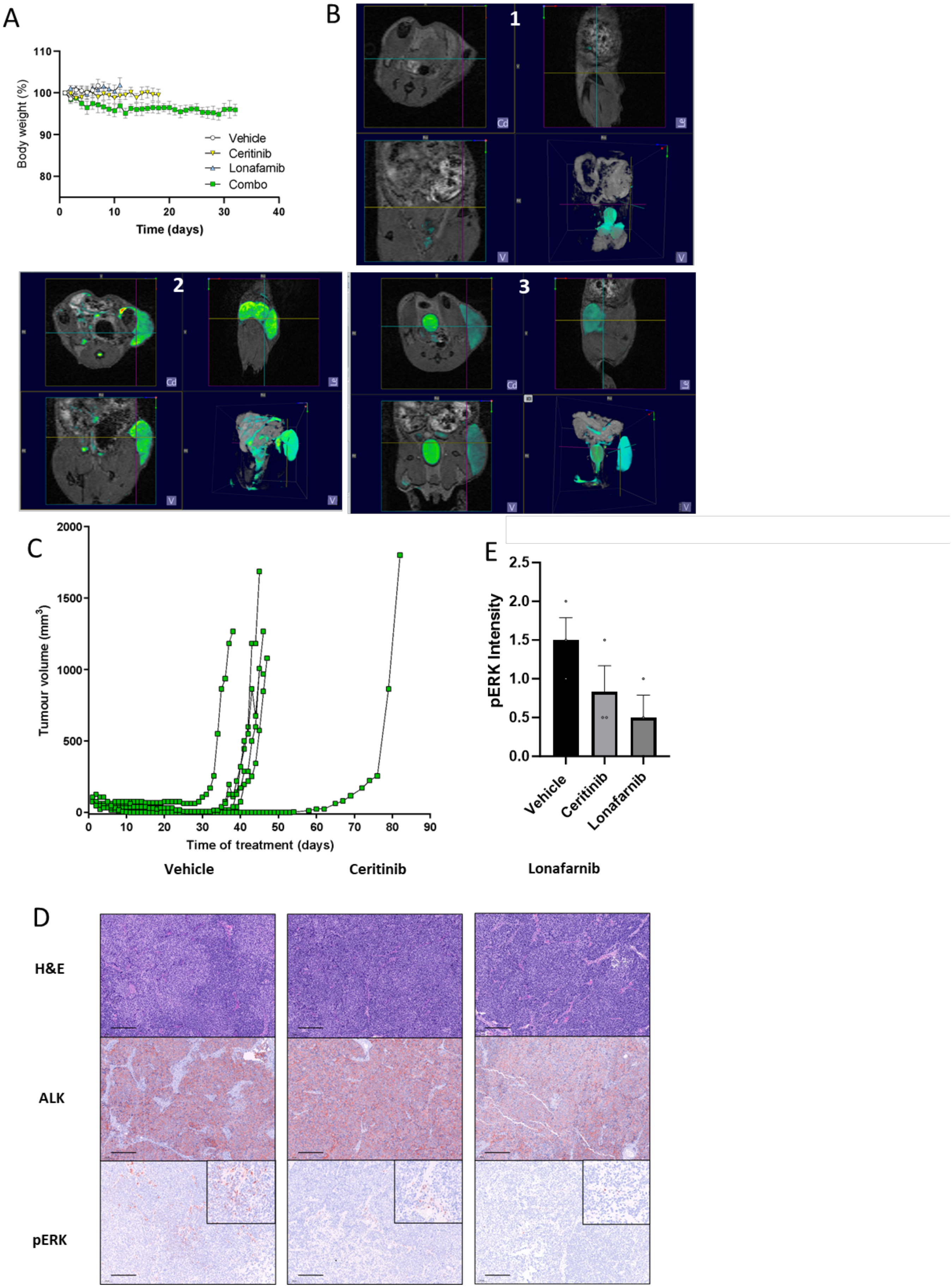
A
Body weight (%)
110
100
90
80
0
10
20
30
40
Time (days)
Vehicle
Ceritinib
Lonafamib
Combo
B
1
2
3
C
Tumour volume (mm^3)
2000
1500
1000
500
0
Time of treatment (days)
E
pERK Intensity
2.5
2.0
1.5
1.0
0.5
0.0
Vehicle
Ceritinib
Lonafarnib
Vehicle
Ceritinib
Lonafarnib
D
H&E
ALK
pERK

Supplementary Figure 17. Combining an ALK inhibitor (ceritinib) with the farnesyltransferase inhibitor lonafarnib significantly reduces tumour growth *in vivo* and lonafarnib effectively inhibits ALK and pERK expression

(**A**) Mouse body weight recorded everyday of treatment relative to baseline weights for each treatment group. Data points (n=6 individual mice) represent means ± SEM. (B) MRI scan from axial (top-left) coronal (bottom-left) sagittal (top-right) and 3D (bottom-right) sections of animals treated with a combination of lonafarnib and ceritinib at the end of treatment (day 30) (1) compared to relapses 9 (2) and 10 (3) days following the end of treatment (days 30+9 and 30+10 respectively). The green shading identifies bladder, testes (mouse 1 is male), vasculature and tumours. (C) Tumour volume over time of NSG mice injected sub-cutaneously with COG-N-426x primary NB cells which reached 75mm^3 before daily administration of ceritinib and lonafarnib (combo), where the new study endpoint is defined as the time taken for tumours to reach 15 mm diameter. Each data point represents one animal. (D) Immunohistochemistry of H&E, ALK and pERK protein of vehicle (20% hydroxypropyl beta cyclodextrin), ceritinib (30 mg/kg) and lonafarnib (40 mg/kg) treated COG-N-426 (FELIX) PDX tumours at the study endpoint (day 30). Magnification bar = 100µm, inserts = 200x magnification. (E) Quantification of phospho-ERK staining intensity from 3 mice per treatment group (with data points shown (n=3)), each with two replicate sections. Source data are provided as a Source Data file.

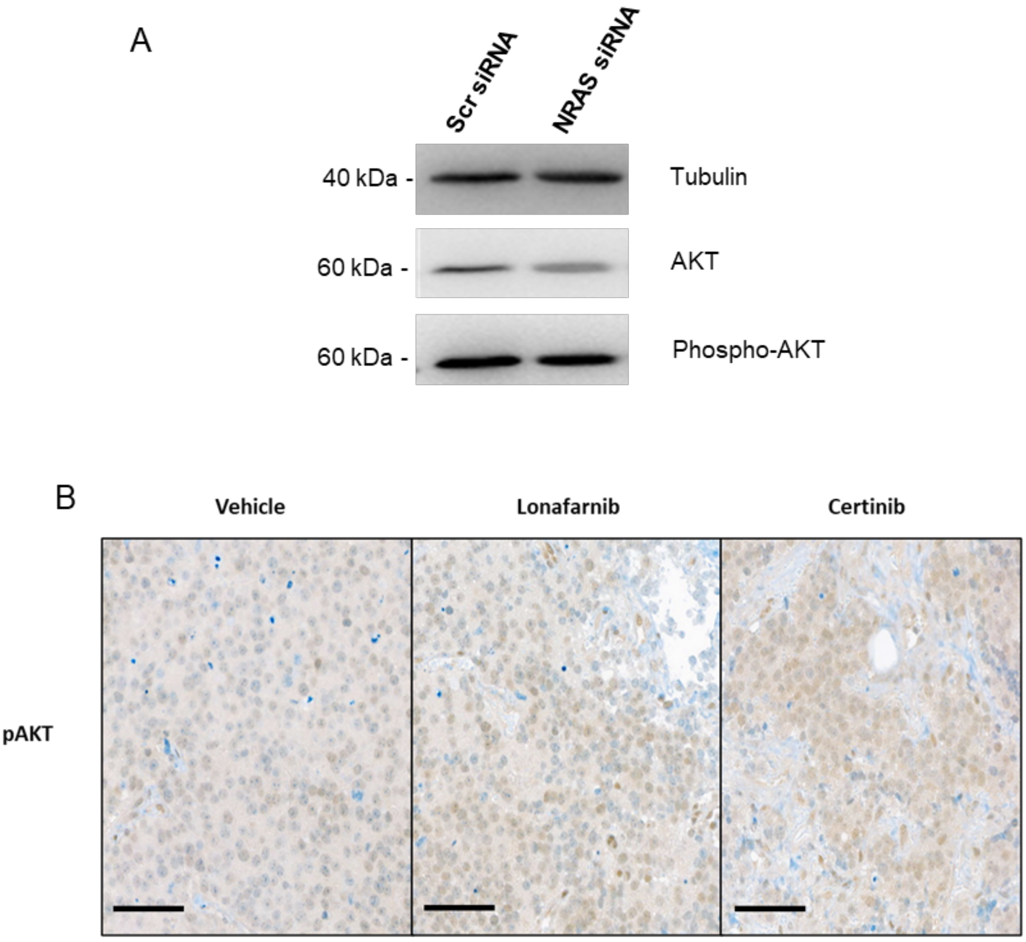

Supplementary Figure 18. ***NRAS*** **targeting via knockdown or pharmacological inhibition does not affect pAKT expression.**

(A) Western blot of the indicated proteins in SHSY5Y cells upon NRAS knockdown. (B) Immunohistochemistry of H&E, ALK and pAKT protein of vehicle (20% hydroxypropyl beta cyclodextrin), ceritinib (30 mg/kg) and lonafarnib (40 mg/kg) treated COG-N-426 (FELIX) PDX tumours at the study endpoint (day 30). Magnification bar = 50µm, inserts = 200x magnification.

miRNA identified by the screen	Genomic location	Family	Other miRNAs in the cluster at the genomic location
Hsa-miR-1304	11q21	MIPF0001064; mir-1304	-
Hsa-miR-136	14q32.2	MIPF0000099; mir-136	hsa-mir-665<br>hsa-mir-431<br>hsa-mir-433<br>hsa-mir-127<br>hsa-mir-432<br>hsa-mir-136
Hsa-miR-4746	19p13.3	-	-
Hsa-miR-7975	19q13.42	-	-

Supplementary Table 1: Genomic location, family to which the miRNA belongs and clustered miRNAs in the region of the top 4 miRNAs (*hsa-mir-136*, *hsa-mir-1304*, *hsa-mir-7975* and *hsa-mir-4746*) identified in at least 3 of the 4 CRISPR GeCKO screens.

Cell line	Source	ALK		*MYCN* copy number	p53
		Nucleotide substitution	Amino acid change		
CHLA-15	COG	3824G>A	1275R>Q	Non-amplified	Functional
CHLA-20	COG	3824G>A	1275R>Q	Non-amplified	Functional
CHLA-90	COG	3733T>G (het)	1245F>V	Non-amplified	Non-functional
CHLA-95	COG	3375C>A (het; syn); 3824G>A (het)	1275R>Q	Amplified	Unknown
CHLA-171	COG	3375C>A (het; syn)		Non-amplified	Non-functional
CHP-134	ECACC			Amplified	Functional
GIMEN	DSMZ			Non-amplified	Functional
KELLY	ECACC	3522C>A	1174F>L	Amplified	Non-functional
LAN-1	ECACC	3375C>A (het; syn); 3522C>A	1174F>L	Amplified	Non-functional
LAN-6	COG			Non-amplified	Functional
NB1643	COG	3824G>A	1275R>Q	Amplified	Functional
NBEBC1	COG	3375C>A (het; syn)		Amplified	Unknown
NBL-S	DSMZ			Non-amplified	Functional
NGP	DSMZ			Amplified	Functional
SH-SY5Y	ECACC	3522C:A (het)	1174F>L	Non-amplified	Functional
SK-N-FI	COG	3375C>A (het; syn); 3408C>T (het; syn)		Non-amplified	Non-functional
SMS-LHN	COG	3375C>A (het; syn)		Non-amplified	Functional

Supplementary Table 2: Attributes of the NB cell line panel

**Sequence ID**	**Cat N#**
hsa-miR-136-5p inhibitor	IH-300629-05-0002
hsa-miR-1304-5p inhibitor	IH-301363-01-0002
hsa-miR-7975 inhibitor	IH-302860-01-0002
hsa-miR-4746-5p inhibitor	IH-302338-01-0002
Negative control 1 inhibitor	IN-001005-01-05
hsa-miR-1304-5p mimics	C-301363-00-0002
Negative control 1 mimics	CN-001000-01-05

Supplementary Table 3: miR INH-mimic sequences

<table>
<tr><th></th><th>Predicted consequential pairing of target region (top) and miRNA (bottom)</th></tr>
<tr><td>Position 1105-1111 of NRAS 3' UTR
hsa-miR-1304-5p</td><td>5' ...UCAGUGAUUUUCAAACCUCAAAU...
||||||
3' GUGUAGAGUGACAUCGGAGUUU</td></tr>
<tr><td>Position 2594-2600 of NRAS 3' UTR
hsa-miR-1304-5p</td><td>5' ...AAGAAUUCUAGUUUACCUCAAAA...
||||||
3' GUGUAGAGUGACAUCGGAGUUU</td></tr>
<tr><th></th><th>Predicted consequential pairing of target region (top) and miRNA (bottom)</th></tr>
<tr><td>Position 1660-1667 of IQGAP1 3' UTR
hsa-miR-1304-5p</td><td>5' ...AUUACAACUAGCAUUGCCUCAAA...
|||||||
3' GUGUAGAGUGACAUCGGAGUUU</td></tr>
<tr><th></th><th>Predicted consequential pairing of target region (top) and miRNA (bottom)</th></tr>
<tr><td>Position 3588-3595 of PTPN11 3' UTR
hsa-miR-1304-5p</td><td>5' ...AAGUUUCAUCCUAAGCCUCAAA...
||| |||||||
3' GUGUAGAGUGACAU-CGGAGUUU</td></tr>
<tr><th></th><th>Predicted consequential pairing of target region (top) and miRNA (bottom)</th></tr>
<tr><td>Position 85-91 of RRAS 3' UTR
hsa-miR-1304-5p</td><td>5' ...UGAGGCCCUCACUGA-GCCUCAAU...
||||||| |||||||
3' GUGUAGAGUGACAUCGGAGUUU</td></tr>
</table>

Supplementary Table 4: TargetScan-predicted binding sites of *miR-1304-5p* to *NRAS*, *RRAS*, *PTPN11* and *IQGAP1* mRNAs.

Supplementary Figures uncropped blots

Extended Data Figure 3F

PARP/cleaved PARP

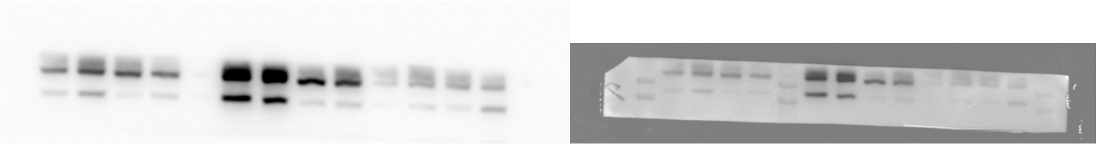

p-ERK

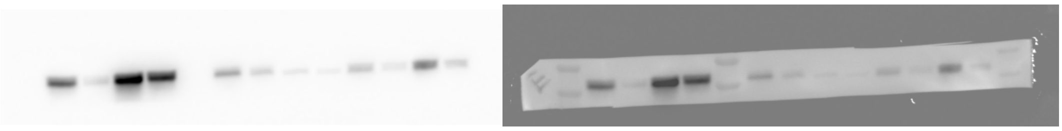

Tubulin

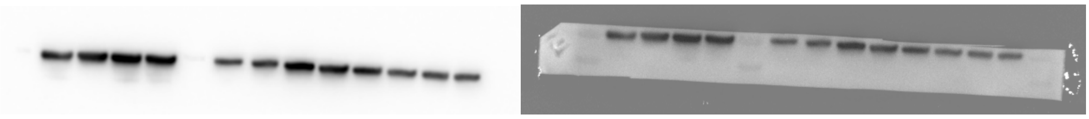

Extended Data Figure 5

p-ERK

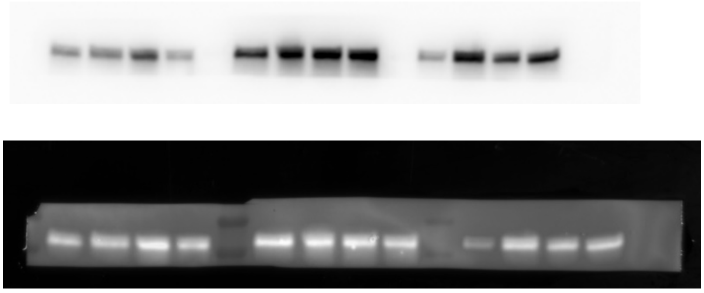

NRAS

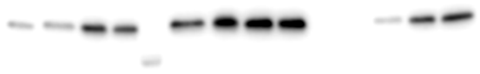

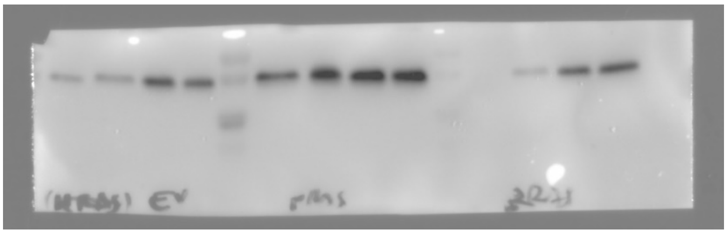

Tubulin

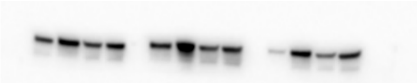

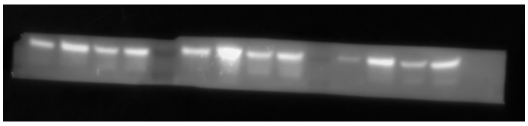

RRAS

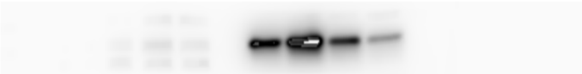

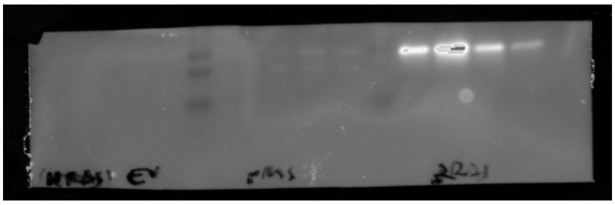

p-ERK

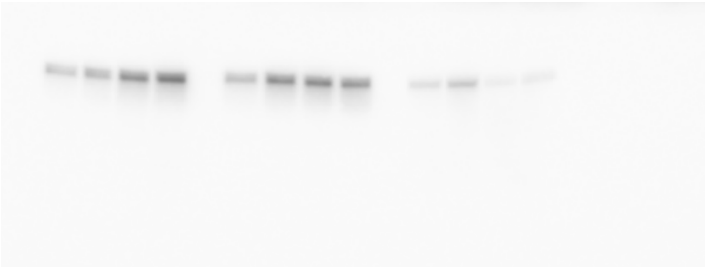

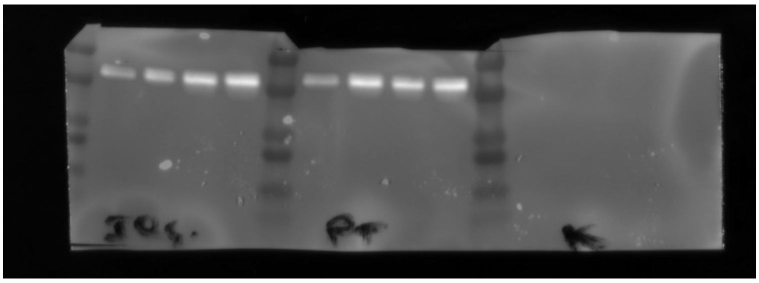

IQGAP1

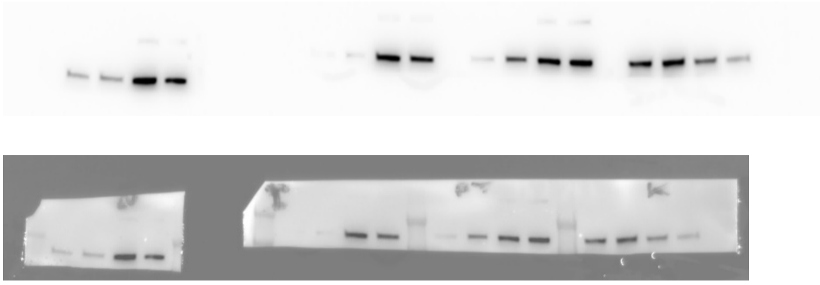

Tubulin

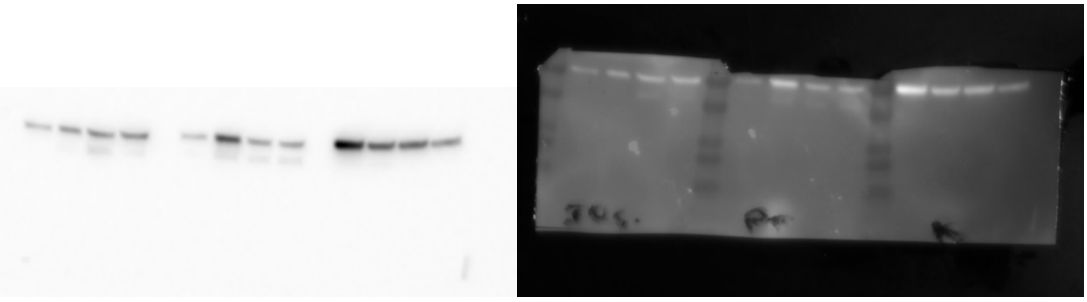

PTPN11

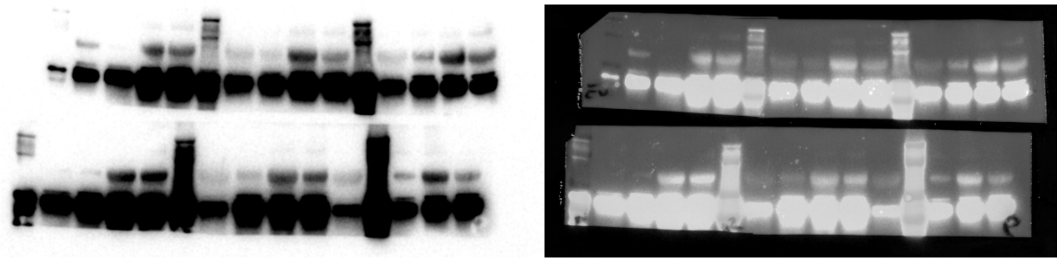

Extended data figure 7

IQGAP1

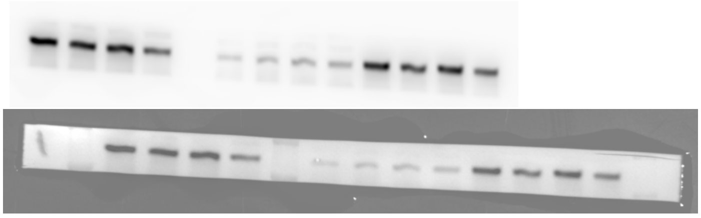

PTPN11

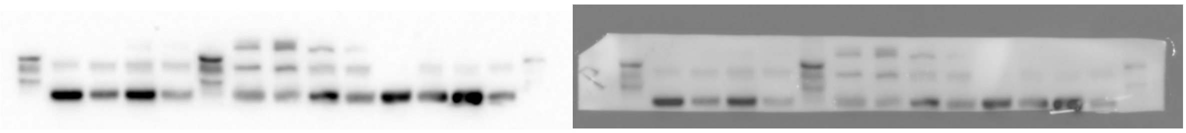

RRAS

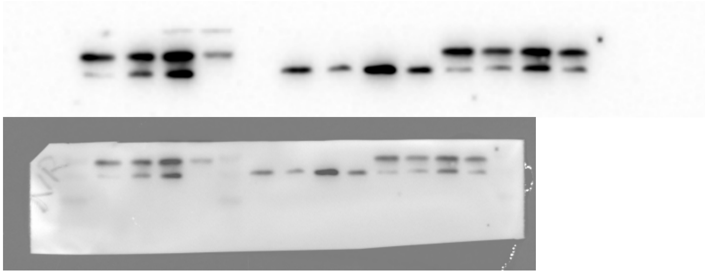

NRAS

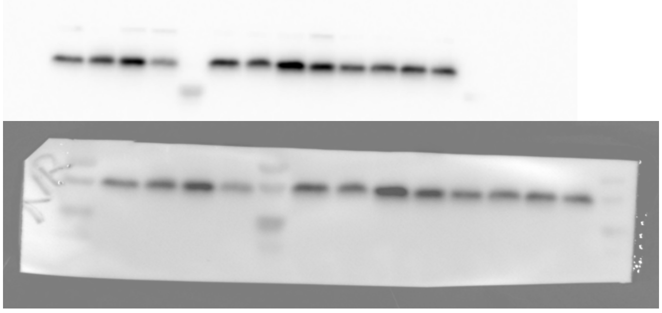

p-ERK

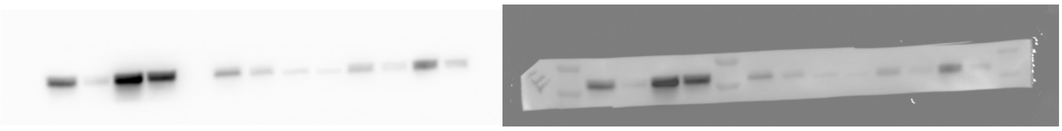

Tubulin

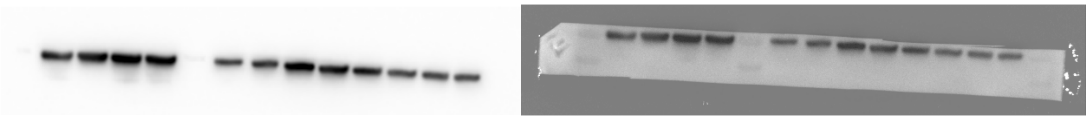

Extended Data Figure 8E

Tubulin

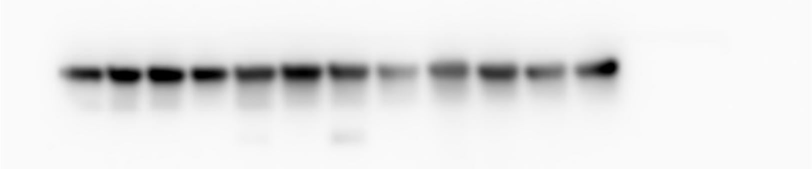

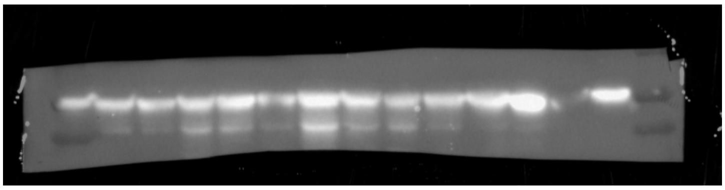

PARP/cleaved PARP

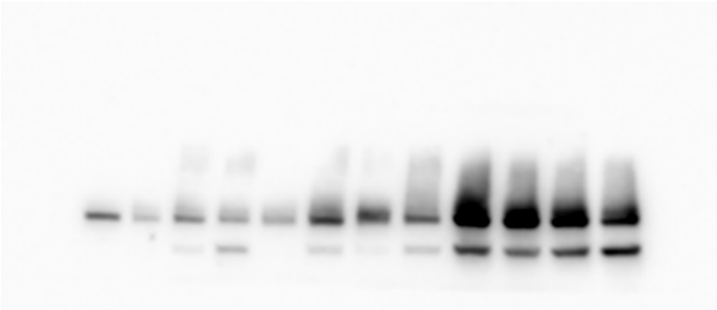

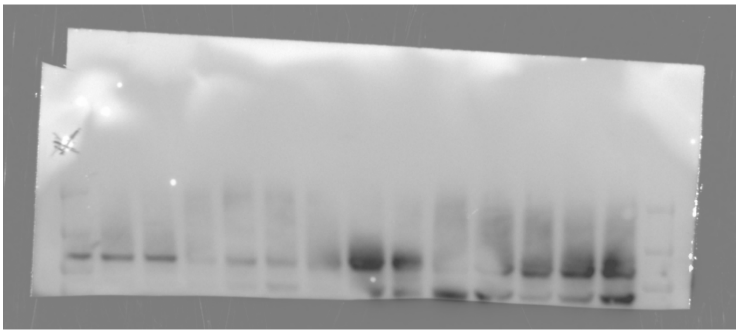

p-ERK

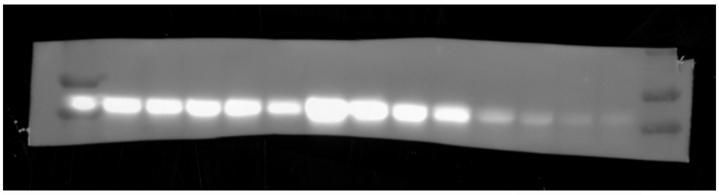

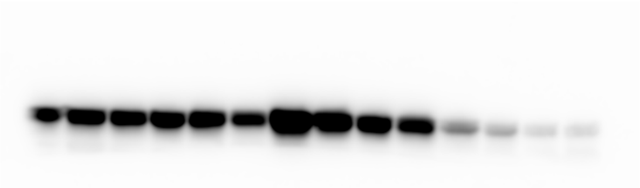

Extended data figure 9B

pan-RAS

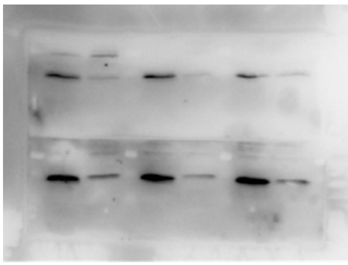

pERK

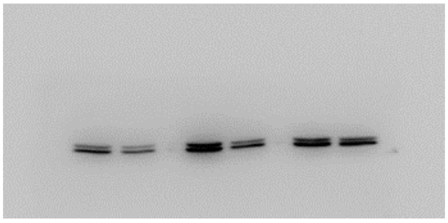

Tubulin

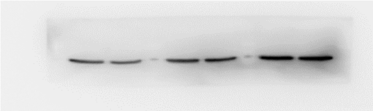

Extended data figure 10D

PARP/cleaved PARP

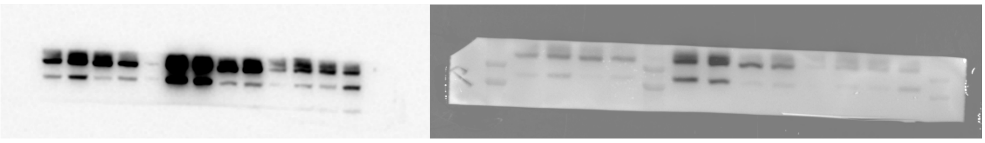

p-ERK

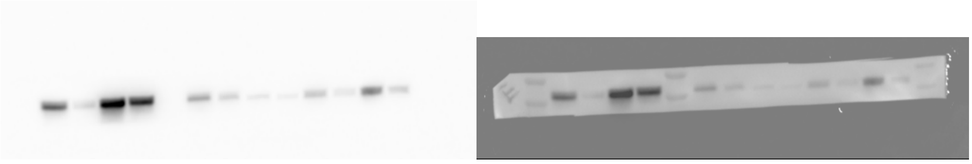

Tubulin

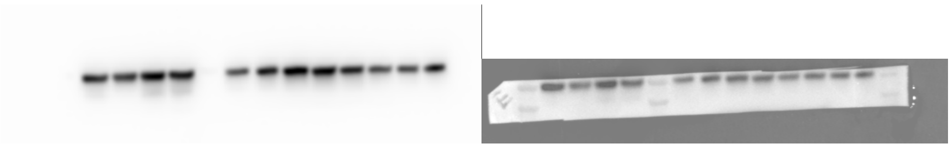

Extended Data Figure 12 E,F

PARP/cleaved PARP

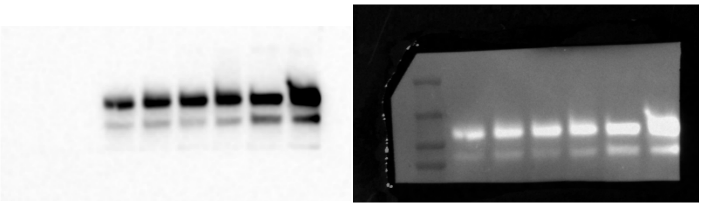

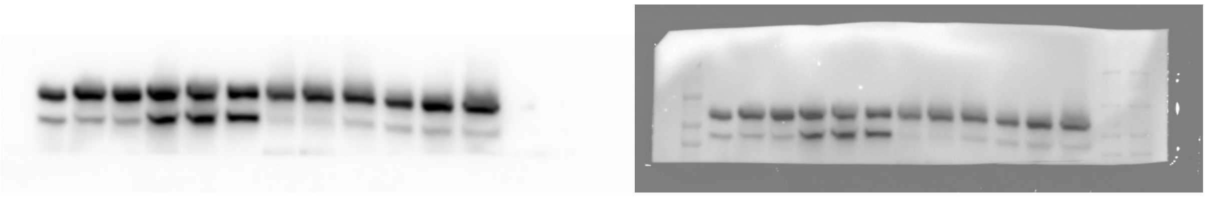

pERK

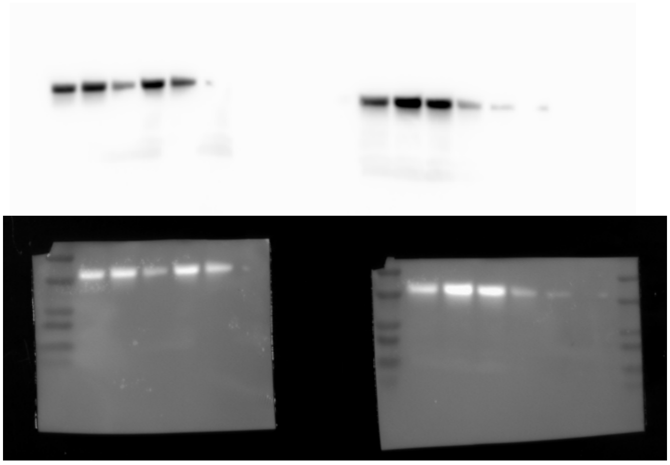

Tubulin

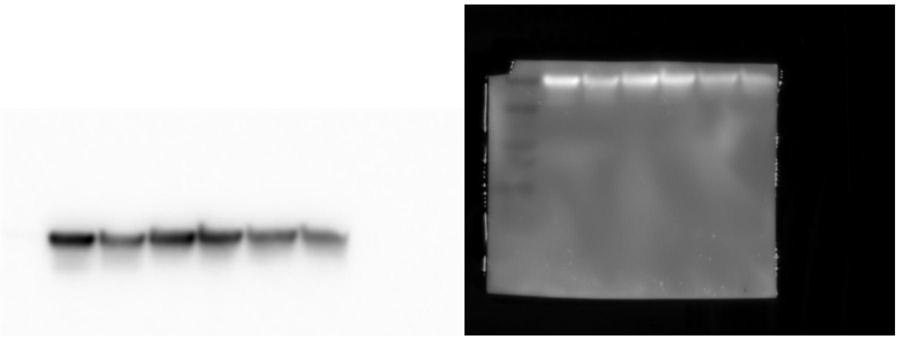

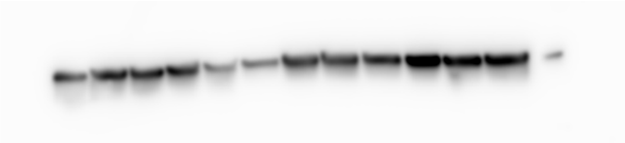

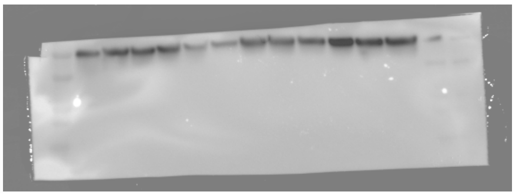

Extended Data Figure 18A

Tubulin

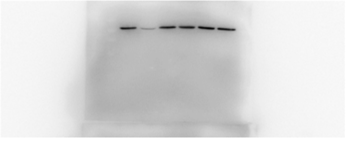

p-AKT
