# Supplementary material for: Targeting NRAS via miR-1304-5p or farnesyltransferase inhibition confers sensitivity to ALK inhibitors in ALK-mutant neuroblastoma
Source: Nat Commun. 2024 Apr 23;15:3422. doi: 10.1038/s41467-024-47771-x (PMC11039739; doi:10.1038/s41467-024-47771-x)
Supplement: Supplementary file 2 — Reporting Summary [file 41467_2024_47771_MOESM2_ESM.pdf]

nature portfolio

Corresponding author(s): Suzanne Turner
Last updated by author(s): 13/02/24

Reporting Summary

Nature Portfolio wishes to improve the reproducibility of the work that we publish. This form provides structure for consistency and transparency in reporting. For further information on Nature Portfolio policies, see our Editorial Policies and the Editorial Policy Checklist.

Statistics

For all statistical analyses, confirm that the following items are present in the figure legend, table legend, main text, or Methods section.

n/a	Confirmed	
☐	☒	The exact sample size (*n*) for each experimental group/condition, given as a discrete number and unit of measurement
☐	☒	A statement on whether measurements were taken from distinct samples or whether the same sample was measured repeatedly
☐	☒	The statistical test(s) used AND whether they are one- or two-sided<br>*Only common tests should be described solely by name; describe more complex techniques in the Methods section.*
☒	☐	A description of all covariates tested
☒	☐	A description of any assumptions or corrections, such as tests of normality and adjustment for multiple comparisons
☐	☒	A full description of the statistical parameters including central tendency (e.g. means) or other basic estimates (e.g. regression coefficient) AND variation (e.g. standard deviation) or associated estimates of uncertainty (e.g. confidence intervals)
☐	☒	For null hypothesis testing, the test statistic (e.g. *F*, *t*, *r*) with confidence intervals, effect sizes, degrees of freedom and *P* value noted<br>*Give P values as exact values whenever suitable.*
☒	☐	For Bayesian analysis, information on the choice of priors and Markov chain Monte Carlo settings
☒	☐	For hierarchical and complex designs, identification of the appropriate level for tests and full reporting of outcomes
☐	☒	Estimates of effect sizes (e.g. Cohen's *d*, Pearson's *r*), indicating how they were calculated

Our web collection on statistics for biologists contains articles on many of the points above.

Software and code

Policy information about availability of computer code

Data collection	Microplate data were collected using SoftmaxPr v7 software. RT-qPCR data were collected using QuantStudio V1.1 software
Data analysis	GraphPad Prism V9 software. FastQC was used to evaluate quality of sequencing reads for CRISPR-Cas9 screen. GSEA Desktop v3 was used to conduct gene set enrichment analysis and cBioportal platform was used to conduct Kaplan-Meier survival analysis of public neuroblastoma patient datasets.

For manuscripts utilizing custom algorithms or software that are central to the research but not yet described in published literature, software must be made available to editors and reviewers. We strongly encourage code deposition in a community repository (e.g. GitHub). See the Nature Portfolio guidelines for submitting code & software for further information.

Data

Policy information about availability of data

All manuscripts must include a data availability statement. This statement should provide the following information, where applicable:

- Accession codes, unique identifiers, or web links for publicly available datasets
- A description of any restrictions on data availability
- For clinical datasets or third party data, please ensure that the statement adheres to our policy

The authors declare that the data supporting the findings of this study are available within the paper and its supplementary information files.
All data are available in the main text or the supplementary materials. PDX models were provided under MTA from the Childhood Cancer Repository maintained by

the Children's Oncology Group (COG). The raw sequencing data generated in this study have been deposited in the NCBI, SRA database under accession code PRJNA903183 [BioSample (24) SRA (24)]. The normalized read counts from the CRISPR GeCKO, the microarray Gene Analysis in SHSY5Y, microarray Gene Analysis in KELLY and the Gene set enrichment analysis (GSEA) 28 data generated in this study are provided in the Supplementary Data file 1-4, respectively. The remaining data are available within the Article, Supplementary Information or Source Data file.

Human research participants

Policy information about studies involving human research participants and Sex and Gender in Research.

Reporting on sex and gender	N/A
Population characteristics	N/A
Recruitment	N/A
Ethics oversight	N/A

Note that full information on the approval of the study protocol must also be provided in the manuscript.

Field-specific reporting

Please select the one below that is the best fit for your research. If you are not sure, read the appropriate sections before making your selection.

☒ Life sciences ☐ Behavioural & social sciences ☐ Ecological, evolutionary & environmental sciences

For a reference copy of the document with all sections, see nature.com/documents/nr-reporting-summary-flat.pdf

Life sciences study design

All studies must disclose on these points even when the disclosure is negative.

Sample size	For mouse PDX studies: groups of 6-8 mice were analysed per treatment cohort to reach a Power of 95% and an error rate of 0.05, while minimizing animal use.
Data exclusions	No data were excluded from this publication
Replication	All experiments were replicated at least twice. We can confirm that all data presented in this publication were reproducible.
Randomization	The groups of mice were randomized into treatment groups.
Blinding	The individuals dosing the mice and recording weight and tumour size did not have knowledge of the predicted results.

Reporting for specific materials, systems and methods

We require information from authors about some types of materials, experimental systems and methods used in many studies. Here, indicate whether each material, system or method listed is relevant to your study. If you are not sure if a list item applies to your research, read the appropriate section before selecting a response.

Materials & experimental systems

n/a	Involved in the study	
☐	☒	Antibodies
☐	☒	Eukaryotic cell lines
☒	☐	Palaeontology and archaeology
☐	☒	Animals and other organisms
☒	☐	Clinical data
☒	☐	Dual use research of concern

Methods

n/a	Involved in the study	
☒	☐	ChIP-seq
☐	☒	Flow cytometry
☐	☒	MRI-based neuroimaging

Antibodies

Antibodies used	Primary antibodies used were as follows: anti-IQGAP1 (1:200; Santa Cruz Biotechnology (SCBT), cat# sc-376021), PTPN11 (1:200; SH-PTP2 B-1, SCBT, cat# sc-7384), anti-p42/44 MAP Kinase (1:1000; Cell Signalling Technology (CST), cat# 9102), anti-pan-Ras (1:200; SCBT, cat# sc-166691), anti-phospho-p42/44 MAP Kinase (Thr202/Tyr204) (1:1000; CST, cat# 9101S), anti-phospho-AKT (1:1000; CST,

cat# 9271) and vinculin (1:200; SCBT, Cat# sc-73614).

Validation

Every CST antibody undergoes rigorous application-specific validation testing customized according to the target and needs of the individual antibody. CST adhere to the Hallmarks of Antibody Validation™, six complementary strategies that can be used to determine the functionality, specificity, and sensitivity of an antibody in any given assay. CST adapted the work by Uhlen, et. al., ("A Proposal for Validation of Antibodies." Nature Methods (2016)) to build the Hallmarks of Antibody Validation, based on their decades of experience as an antibody manufacturer and their dedication to reproducible science.

SCBT antibodies were validated in multiple publications according to the product datasheets available from the website:

Anti I1GAP1
1. Zhao, H., et al. 2014. Coexpression of IQ-domain GTPase-activating protein 1 (IQGAP1) and dishevelled (Dvl) is correlated with poor prognosis in non-small cell lung cancer. PLoS ONE 9: e113713.
2. Akula, M.K., et al. 2019. Protein prenylation restrains innate immunity by inhibiting Rac1 effector interactions. Nat. Commun. 10: 3975.
3. Sheen, Y.S., et al. 2020. Purpuric drug eruptions induced by EGFR tyrosine kinase inhibitors are associated with IQGAP1-mediated increase in vascular permeability. J. Pathol. 250: 452-463.
4. Negretti, N.M., et al. 2021. The Campylobacter jejuni CiaD effector co-opts the host cell protein IQGAP1 to promote cell entry. Nat. Commun. 12: 1339.

AntiSHP2
1. Tanowitz, M., et al. 1999. Regulation of neuregulin-mediated acetylcholine receptor synthesis by protein tyrosine phosphatase SHP2. J. Neurosci. 19: 9426-9435.
2. Buonato, J.M., et al. 2015. EGF augments TGFb-induced epithelial-mesenchymal transition by promoting SHP2 binding to GAB1. J. Cell Sci.
128: 3898-3909.
3. Xu, S., et al. 2016. PECAM1 regulates flow-mediated Gab1 tyrosine phosphorylation and signaling. Cell. Signal. 28: 117-124.

Anti pan-Ras
1. Tamburini, B.A., et al. 2010. Gene expression profiling identifies inflammation and angiogenesis as distinguishing features of canine hemangiosarcoma. BMC Cancer 10: 619.
2. Yang, G., et al. 2013. RAS promotes tumorigenesis through genomic instability induced by imbalanced expression of Aurora-A and BRCA2 in midbody during cytokinesis. Int. J. Cancer 133: 275-285.
3. Zaganjor, E., et al. 2014. Ras transformation uncouples the kinesincoordinated cellular nutrient response. Proc. Natl. Acad. Sci. USA 111:
10568-10573.
4. Zhang, W., et al. 2015. Adiponectin affects vascular smooth muscle cell proliferation and apoptosis through modulation of the mitofusin-2-mediated Ras-Raf-Erk1/2 signaling pathway. Mol. Med. Rep. 12: 4703-4707.
5. Siprashvili, Z., et al. 2016. The noncoding RNAs SNORD50A and SNORD50B bind K-Ras and are recurrently deleted in human cancer. Nat. Genet. 48: 53-58.
6. Ahn, S.Y., et al. 2017. Anti-helminthic niclosamide inhibits Ras-driven oncogenic transformation via activation of GSK-3. Oncotarget 8: 31856-31863.
7. Hagiwara, N., et al. 2018. Mevalonate pathway blockage enhances the efficacy of mTOR inhibitors with the activation of retinoblastoma protein in renal cell carcinoma. Cancer Lett. 431: 182-189.
8. Che, Y., et al. 2019. KRAS regulation by small non-coding RNAs and SNARE proteins. Nat. Commun. 10: 5118.

Anti-vinculin
1. Palmieri, D., et al. 2011. HMGA proteins promote ATM expression and enhance cancer cell resistance to genotoxic agents. Oncogene 30: 3024-3035.
2. Zhang, T., et al. 2013. The contributions of HIF-target genes to tumor growth in RCC. PLoS ONE 8: e80544.
3. Guo, Q., et al. 2014. PAK4 kinase-mediated SCG10 phosphorylation involved in gastric cancer metastasis. Oncogene 33: 3277-87.
4. Yuzugullu, H., et al. 2015. A PI3K p110β-Rac signalling loop mediates Pten-loss-induced perturbation of haematopoiesis and leukaemogenesis. Nat. Commun. 6: 8501.
5. Cataldo, A., et al. 2016. MiR-302b enhances breast cancer cell sensitivity to cisplatin by regulating E2F1 and the cellular DNA damage response. Oncotarget 7: 786-797.

6. Hoppe-Seyler, K., et al. 2017. Induction of dormancy in hypoxic human papillomavirus-positive cancer cells. Proc. Natl. Acad. Sci. USA 114: E990-E998.
7. Avolio, R., et al. 2018. Protein Syndesmos is a novel RNA-binding protein that regulates primary cilia formation. Nucleic Acids Res. 46: 12067-12086.
8. Schnack, L., et al. 2019. Mechanisms of trained innate immunity in oxLDL primed human coronary smooth muscle cells. Front. Immunol. 10: 13.
9. Ballabio, C., et al. 2020. Modeling medulloblastoma in vivo and with human cerebellar organoids. Nat. Commun. 11: 583.
10.Hollenbach, M., et al. 2021. Pitfalls in AR42J-model of cerulein-induced acute pancreatitis. PLoS ONE 16: e0242706.

Eukaryotic cell lines

Policy information about cell lines and Sex and Gender in Research

Cell line source(s)

The neuroblastoma cell lines CHLA-15, CHLA-20, CHLA-90, CHLA-95, CHLA-171, COG-N-426 (Felix), COG-N-415, COG-N-557, LA-N-5, LA-N-6, NB-1643, NB-EBC1, SK-N-FI, and SMS-LHN were obtained from the Children's Oncology Group Childhood Cancer Repository. CHP-134, KELLY, LA-N-1 and SH-SY5Y were obtained from the European Collection of Authenticated Cell Cultures. GI-ME-N, NBL-S and NGP were obtained from German Collection of Microorganisms and Cell Cultures and 293FT was obtained from Thermo Fisher Scientific.

Authentication

All cells were autenticated by STR profiling at the repositories.

Mycoplasma contamination

All cells were tested quarterly for Mycoplasma contamination during the project and were negative.

Commonly misidentified lines
(See ICLAC register)

None

Animals and other research organisms

Policy information about studies involving animals; ARRIVE guidelines recommended for reporting animal research, and Sex and Gender in Research

Laboratory animals

Nod SCID Gamma mice (Charles River) were used. Ages ranged from 6-8 weeks at the beginning of the study and were a random mix of females and males. NSG mice were housed in groups of 2–6 mice per cage in individually ventilated cages with a 12 hour light/dark cycle.

Wild animals

No wild animals were used in this study.

Reporting on sex

The same number of males and females were used in each study.

Field-collected samples

No field-collected samples were used in the study.

Ethics oversight

Ethical approval was sought and obtained from the University of Cambridge Animal Welfare and Ethical Review board (AWERB). All experiments were conducted in accordance with the Animal (Scientific Procedures) Act 1986 under the Project Licence P4DBEFF63

Note that full information on the approval of the study protocol must also be provided in the manuscript.

Flow Cytometry

Plots

Confirm that:

☒ The axis labels state the marker and fluorochrome used (e.g. CD4-FITC).

☒ The axis scales are clearly visible. Include numbers along axes only for bottom left plot of group (a 'group' is an analysis of identical markers).

☐ All plots are contour plots with outliers or pseudocolor plots.

☒ A numerical value for number of cells or percentage (with statistics) is provided.

Methodology

Sample preparation

Approximately 500,000 SH-SY5Y or KELLY cells were collected by centrifugation following trypsinization, washed with cold PBS, resuspended in 300 µl of cold PBS and fixed by the dropwise addition of 700 µL of 70% ice-cold ethanol (Sigma-Aldrich). Cells were fixed for 30 minutes on ice, then washed twice with ice-cold PBS before resuspending in 100µg/mL RNAse (Sigma-Aldrich) for 30 minutes at 37°C. Propidium iodide (PI; 50 µg/mL, Sigma-Aldrich) was added to the cells which were analyzed using a FACS Accuri™ C6 Plus Flow Cytometer (BD Biosciences). Single cells were gated using the FL2-area and –width

	parameters and a minimum of 10,000 events were collected per sample. Data analysis was conducted with FlowJo software (Treestar).
Instrument	BD Accurri C6 flow cytometer
Software	FlowJo V10 Software
Cell population abundance	N/A
Gating strategy	Cells were gated according to physical parameters in order to discard cell debris (FCS/SSC) and cells clumps (Width/Area). Healthy cells were excluded by selecting the PI positive population.

☒ Tick this box to confirm that a figure exemplifying the gating strategy is provided in the Supplementary Information.

Magnetic resonance imaging

Experimental design

Design type	Magnetic resonance imaging (MRI) data was acquired using a 3T BioSpec Bruker system (Ettlingen, Germany) with a 40 mm quadrature volume coil. Animals were anaesthetised with isoflurane (induction 3%, maintenance 2%) in 100% oxygen, adjusted thereafter to normalise respiration rate, which was maintained at 40-60 breaths per minute using a pneumatic pillow (ERT Control Gating Module, SA Instruments, New York, United States). Animals were positioned in an MRI compatible cradle fitted with a heated air supply (Thermo Fisher Scientific, Massachusetts, United States), allowing the temperature to be maintained at 35-36 °C using a rectal probe. Anatomical 3D FISP sagittal images were acquired with a scan time of 4 m 28 s, with the following parameters: field-of-view (FOV) = 30 × 30 × 30 mm3, matrix = 128 × 128 × 64, TR = 6.5 ms, TE = 3 ms, bandwidth = 37 kHz, averages = 3, with a flip angle of 15 degrees and eight segments. For tumoural contrast, sagittal 3D T2-weighted images were acquired with turboRARE and the following parameters: TE = 84 ms, TR = 1200 ms, averages =1, scan time = 7 m 40 s, echo spacing 12 ms, RARE factor = 16, FOV = 30 × 30 × 30 mm3, matrix size = 100 × 100 × 64, bandwidth = 20.8 kHz, with fat suppression. Following image acquisition animals were placed in a recovery box on a heated pad set to 37 °C. Images were viewed in ParaVision 360 V2.0 with the T2-weighted image (green) overlaid onto the anatomical 3D FISP image (grey), which then allowed for 3D visualisation and high contrast.
Design specifications	There were four imaging sessions. Each animal subject was imaged once, as part of only this trial, with 3D FISP lasting 4 min 28 sec and T2-weighted imaging lasting 7 min 40 seconds. Additional imaging was performed for setup, localization, and in three orthogonal views to ensure that we captured visualisation of the tumor. The entire protocol occurred in under an hour.
Behavioral performance measures	No behavioral performance measures were used.

Acquisition

Imaging type(s)	Anatomical 3D FISP sagittal images and sagittal 3D T2-weighted images
Field strength	3 tesla
Sequence & imaging parameters	For the anatomical FISP: Field-of-view (FOV) = 30 × 30 × 30 mm3, matrix = 128 × 128 × 64, TR = 6.5 ms, TE = 3 ms, bandwidth = 37 kHz, averages = 3, with a flip angle of 15 degrees and eight segments, scan time = 4 m 28 s.<br><br>For the T2-weighted image for high tumor contrast: FOV = 30 × 30 × 30 mm3, matrix size = 100 × 100 × 64, TR = 1200 ms, TE = 84 ms, averages =1, scan time = 7 m 40 s, echo spacing 12 ms, RARE factor = 16, bandwidth = 20.8 kHz, with fat suppression.
Area of acquisition	The area of acquisition was between the base of the spine to just above the kidneys. This was considered to be sufficient coverage to encapsulate the entire tumor volume.

Diffusion MRI ☐ Used ☒ Not used

Preprocessing

Preprocessing software	ParaVision 360 V2.0
Normalization	Quantitative morphological parameters were measured, which did not require normalization. The tumor had sufficiently high contrast against the background tissue to not require any normalization.
Normalization template	Quantitative morphological parameters were measured, which did not require normalisation.
Noise and artifact removal	There was no structured noise, and the animal was imaged free-breathing.
Volume censoring	Paravision, provided by Bruker, allowed visualization of the T2-weighted signal on overlaid on top of the anatomical FISP image. This allowed for direct visualization of the tumor, which consisted of any hyperintensity greater than 50% of the mean signal that was subcutaneous near the flank of the mouse. This high contrast allowed for a direct measurement of the tumor volume.

Statistical modeling & inference

Model type and settings	No advanced statistical model was used for the MRI data, as simple reporting of the presence, growth, or shrinkage of the tumors was sufficient to show the effects of the drug, especially in the presence of the other data presented in the paper.
Effect(s) tested	There were no stimulus used.<br><br>Morphological changes, specifically, the presence of tumor, its growth or shrinkage, were reported:<br>"MRI analysis was performed after 30 days of daily treatment and showed that one of the mice had no detectable tumour although the others had residual tumours of 1-6mm diameter (Fig. 7D). However, 2 mice with small palpable tumours from the combination treatment group analysed at day 9 and 10 following cessation of treatment showed tumour progression (Fig. 7D, Extended Data Fig. 9A)."

Specify type of analysis: ☐ Whole brain ☒ ROI-based ☐ Both

Anatomical location(s)	The regions were pseudo-automatically defined: the region of the bladder had hyperintensity, but was excluded from analysis; the region of the tumor was all of the bright T2-weighted signal that was subcutaneous at the site of the injection and abnormal, which could be
Statistic type for inference (See Eklund et al. 2016)	N/A
Correction	No correction was used for the MRI data

Models & analysis

n/a	Involved in the study
☒	☐ Functional and/or effective connectivity
☒	☐ Graph analysis
☒	☐ Multivariate modeling or predictive analysis
