# Supplementary material for: Targeting NRAS via miR-1304-5p or farnesyltransferase inhibition confers sensitivity to ALK inhibitors in ALK-mutant neuroblastoma
Source: Nat Commun. 2024 Apr 23;15:3422. doi: 10.1038/s41467-024-47771-x (PMC11039739; doi:10.1038/s41467-024-47771-x)
Supplement: Supplementary file 4 — Description of Additional Supplementary Files [file 41467_2024_47771_MOESM4_ESM.pdf]

Description of Additional Supplementary Files

Supplementary Data 1. Analysis of SH-SY5Y GeCKO CRISPR screen sequencing data by k-means clustering showing common but also divergent genes/microRNAs that on KO confer a survival advantage to cells at different concentrations (300 and 750nM) of ALK TKIs (brigatinib and ceritinib). For each treatment condition, an arbitrary threshold (>1.8) of fold difference (FD) in gene enrichment between DMSO and ALK TKI treatment was set, and the genes with multiple sgRNAs exceeding the FD threshold were considered to be significantly enriched.

Supplementary Data 2. Raw signal data and statistics (t-test) from the genome-wide expression microarray (HT-12 v4) analysis performed on SH-SY5Y cells transfected with a *miR-1304-5p* mimic or a scrambled negative control.

Supplementary Data 3. Raw signal data and statistics (t-test) from the genome-wide expression microarray (HT-12 v4) analysis performed on KELLY cells transfected with a *miR-1304-5p* mimic or a scrambled negative control.

Supplementary Data 4. Gene set enrichment analysis (GSEA) performed with MSigDB's 'hallmark gene sets' to identify the molecular mechanisms driving the cellular phenotype induced by *miR-1304-5p*. Genome-wide expression microarray (HT-12 v4) analysis was performed on SH-SY5Y and KELLY cells transfected with a *miR-1304-5p* mimic or a scrambled negative control, detecting 1267 and 1103 altered genes in SH-SY5Y and KELLY cell lines, respectively (SHSY5Y all and KELLY all sheets, respectively). Sheets "positive NES KELLY" and "positive NES SH" show the genes with a positive NES score and highlight the 6 gene sets identified that were significantly (p<0.05) altered following miR-1304-5p transfection with a negative correlation: xenobiotic_metabolism, epithelial_mesenchymal_transition, estrogen_response_late, interferon_gamma_response, glycolysis, kras_signaling and mtorc1_signaling.
